# Comparative Phylogeography of Two Specialist Rodents in Forest Fragments in Kenya

**DOI:** 10.3390/life14111469

**Published:** 2024-11-12

**Authors:** Alois Wambua Mweu, Kenneth Otieno Onditi, Laxman Khanal, Simon Musila, Esther Kioko, Xuelong Jiang

**Affiliations:** 1 Key Laboratory of Genetic Evolution and Animal Models, Kunming Institute of Zoology, Chinese Academy of Sciences, Kunming 650201, China; aliwambua@gmail.com; 2 Zoology Section, National Museums of Kenya, Nairobi P.O. Box 40658-00100, Kenya; 3 Sino-Africa Joint Research Centre, Chinese Academy of Sciences, Nairobi P.O. Box 62000-00200, Kenya; 4 Central Department of Zoology, Institute of Science and Technology, Tribhuvan University, Kathmandu 44618, Nepal; lkhanal@cdztu.edu.np

**Keywords:** *Praomys jacksoni*, *Hylomyscus endorobae*, phylogeography, genetic diversity, habitat fragmentation, tropical forests

## Abstract

The fragmented forests of the Kenya highlands, known for their exceptional species richness and endemism, are among the world’s most important biodiversity hotspots. However, detailed studies on the fauna of these ecosystems—especially specialist species that depend on moist forests, which are particularly threatened by habitat fragmentation—are still limited. In this study, we used mitochondrial genes (cytochrome b and the displacement loop) and a nuclear marker (retinol-binding protein 3) to investigate genetic and morphological diversity, phylogenetic associations, historical divergence, population dynamics, and phylogeographic patterns in two rodent species—the soft-furred mouse (*Praomys jacksoni*) and the African wood mouse (*Hylomyscus endorobae*)—across Kenya’s forest landscapes. We found a complex genetic structure, with *P. jacksoni* exhibiting greater genetic diversity than *H. endorobae*. The Mt. Kenya *P. jacksoni* populations are significantly genetically different from those in southwestern forests (Mau Forest, Kakamega Forest, and Loita Hills). In contrast, *H. endorobae* presented no observable biogeographic structuring across its range. The genetic diversity and geographic structuring patterns highlighted selectively strong effects of forest fragmentation and differing species’ ecological and evolutionary responses to these landscape changes. Our findings further underscore the need for expanded sampling across Kenya’s highland forests to better understand species’ changing diversity and distribution patterns in response to the impacts of human-mediated habitat changes. These insights are critical for informing conservation strategies to preserve biodiversity better in this globally important region.

## 1. Introduction

Human-mediated habitat fragmentation, often resulting from agricultural expansion, urban development, and infrastructure projects, alters species traits, genetic diversity, and community structure [1,2,3]. While forests are crucial biodiversity reservoirs, their ecological integrity is increasingly compromised by human-mediated disturbances, which, in the process, reduce the habitat quality and availability, posing a significant threat to the species dependent on them [4,5]. Species with narrow tolerances to particular climate regimes and human interaction levels, such as forest specialists, have greater vulnerability to increasingly fragmentary forest habitats. There is a need for finer-scale studies into the nature and dynamics of the genetic and phenotypic consequences of habitat fragmentation, which are key to effective strategies to mitigate its effects.

For species with distributions relatively restricted to particular habitats, such as forests, the evolution of specialized traits that optimize survival and reproduction in a narrow niche, while advantageous under stable conditions, becomes a disadvantage when those conditions are unnaturally altered [6]. These species are likely to face more elevated habitat contraction as warming climates force them to small, increasingly limited suitable habitats, such as mountain peaks [7].

Studies have shown that the evolutionary history of most species found in East and Central Africa’s montane regions is underlain by a complex interplay of forest isolation [8,9,10,11], fragmentation, and reconnection of faunal biodiversity, driving increased speciation, population expansion, and contraction [12,13,14]. However, exactly how these processes are related to the current patterns of species diversity and distribution remains poorly understood. The vertebrate distribution patterns in these regions were significantly impacted by late Miocene and Plio-Pleistocene climate conditions, which drove habitat expansion and contraction for most species [8,10,11,15,16,17,18,19,20]. More studies on the contemporary genetic and phenotypic characteristics of fauna in remaining forest patches could enhance our understanding of how forest habitat loss over time influences the pace and direction of evolutionary divergence or convergence.

Habitat fragmentation generally compromises the long-term viability of species by isolating populations and disrupting gene flow, which reduces effective population sizes and diminishes genetic diversity [3], as the disruption of dispersal and ecosystem connectivity limits the ability of species to find mates, access resources, and recolonize niches, ultimately threatening their survival and the resilience of entire ecosystems [1,2,3,4,5]. Global patterns of genetic structuring in fragmented ecosystems show that habitat fragmentation often leads to isolated populations with limited gene flow, increasing genetic differentiation among fragments [1,2,3,4]. These genetic consequences are pronounced in biodiversity-rich regions such as tropical forests, where biodiversity patterns can highly contrast across relatively limited landscapes. Notably, the impacts of contemporary forest fragmentation on most species remain unstudied, with few studies focusing on correlations between species genetics and phenotypes and fragmentation, particularly among Afrotropical forest fauna [12,13,21,22].

In this study, we explored whether species from different forest fragments display systematic variation patterns in genetic and phenotypic characteristics using Jackson’s soft-furred mouse *Praomys jacksoni* (de Winton, 1897) [Rodentia; Muroidea; Muridae; Murinae; *Praomys*] and the Mount Kenya wood mouse *Hylomyscus endorobae* (Heller, 1910) [Rodentia; Muroidea; Muridae; Murinae; *Hylomyscus*] across forest fragments in Kenya [23,24,25,26] as the study model. These species exemplify montane fauna that historically evolved to specialize in cool climates in the Kenyan highlands [27], and despite being abundant in their ranges, their taxonomy remains debated. Recent studies indicate that *P. jacksoni* needs taxonomic reevaluation to better account for gene pools that appear to have accumulated species-level divergences [17], whereas several undescribed *Hylomyscus* lineages were recently highlighted or described as new to science [18,28]. *Praomys jacksoni* is a widespread and abundant species distributed in and around the Congolian forests, with eastern limits in the Kenya highlands. On the other hand, Nicolas et al. [18] identified as many as ten *Hylomyscus* taxa as new lineages or elevated from synonymy, of which Kerbis Peterhans et al. [28] described four lineages as new species, diverged in the late Miocene (e.g., the split between the *denniae* group and sister clades). We examined the phylogeographic patterns, genetic diversity, and speciation processes of *Praomys jacksoni* and *Hylomyscus endorobae* across Kenya’s forests to understand the effects of habitat fragmentation on tropical montane ecosystems and to inform conservation strategies that could be effective in similarly fragmented landscapes. The main hypothesis was that habitat fragmentation has led to distinct phylogeographic structuring and genetic and morphological differentiation patterns within and between species.

## 2. Materials and Methods

### 2.1. Study Area

**Figure 1:**
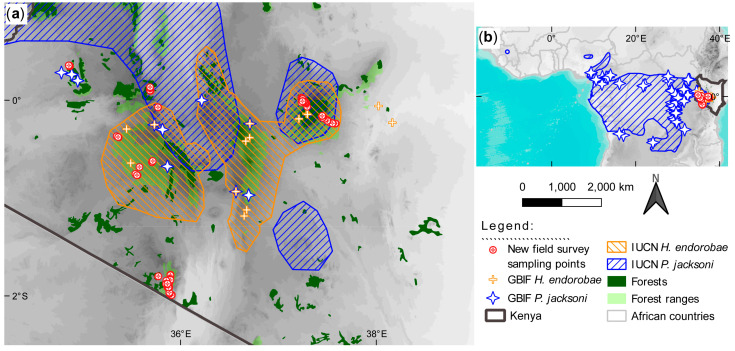
Distribution maps of *Praomys jacksoni* and *Hylomyscus endorobae* across their known distributions*;* (**a**) shows the field survey sampling sites in Kenya [red-outlined circles with a red plus sign] and (**b**) shows the occurrences of these species based on the International Union for Conservation of Nature (IUCN) Red List ranges and geolocated point-occurrence records from the Global Biodiversity Information Facility (GBIF). Grayscale shading in both plots represents elevation with darker shades corresponding to higher elevations. The blue shade in ‘b’ represents the ocean. See the legend for other labeling details.

The *Praomys jacksoni* and *Hylomyscus endorobae* samples used in this study were sourced from fieldwork conducted in Kenya by joint zoological survey teams from the Kunming Institute of Zoology (KIZ) and the National Museums of Kenya (NMK) between 2015 and 2018 (Figure 1). Additionally, we scoured the literature and obtained additional data on these two species from western Kenya Mt. Elgon and Cherangani Hills, the Aberdares Ranges, and the Mau Forest Fragments, thus bridging geographical coverages in sampling critical montane highland ecosystems across the known range of these two lineages in Kenya. Site selection was conducted following reconnaissance surveys in preceding years and transects refined accordingly based on actual field conditions. Key orographic events and geomorphological features characterize these study areas [8,9,29,30,31,32,33,34,35,36], making them classical models for this type of study. A detailed geographical, climatic, and biodiversity characterization of the study sites is provided in Supplementary File S1.

### 2.2. Field Sampling Design

We used Sherman live traps, snap traps, and pitfalls for animal trapping in the field. The trap stations were designed to optimize onsite heterogeneity and, thus, increase the chances of targeting all the small mammals inhabiting the sites. For example, at Mt. Kenya, trap stations were established at different elevation levels, whereas at the other sites, all unique habitats were traversed. Traps were set at the most likely microhabitats where the animals were suspected to occur, such as active burrows, under tree logs, and in defined trail networks with dense vegetation and thick grass. Each trap line had 25–30 trapping stations, with the Sherman and Snaps traps set at approximately 10–15-m intervals. The traps were baited with oat flakes and a single peanut. The traps were inspected every morning and rebaited at the same trapline for two trap nights before moving to the next trapline. For each sample, measurements of the tail, head and body, hindfoot, ear length, and weight of the individuals were recorded in the field before vouchers, either wholly preserved or in part as wet collections in 70% ethanol, were prepared. The selected samples were used as museum samples and muscle and liver tissues were collected in absolute ethanol-filled 2 mL sampling vials for DNA-based molecular studies. See the associated publications for other details on the field sampling protocols—[20,37,38]. The tissues and vouchers are stored in the Mammalogy Section Laboratory at the National Museums of Kenya.

Animal handling procedures adhered to Kenya’s regulations for wildlife research as well as the conventional guiding principles for using wild animals in scientific research [39,40]. The Kenyan fieldwork surveys were approved by the Kenya Wildlife Service Research and Ethics Committee.

### 2.3. DNA Extraction, Amplification, Sequencing, and Sequence Alignment

We extracted total DNA using the sodium dodecyl sulfate procedure [41]. From the total DNA, 1.5 μL of template was PCR-amplified; the total PCR volume of 20 μL of reaction reagents per sample comprised 0.5 μL of gene-specific primers, 10 μL of 2× Power TaqPCR Master Mix, and 8.5 μL of ultrapure water. The PCR settings and primers used for amplifying these genes are summarized in Table A1. We selected and sequenced three genes to ensure robust phylogenetic and demographic analysis: the mitochondrial cytochrome b gene (Cytb), the mitochondrial displacement loop (D-loop), and the nuclear interstitial retinoid-binding protein-encoding gene (RBP3). These genes have been widely utilized in mammalian phylogenetic and population structure studies because of their phylogenetic informativeness and data availability. The Cytb gene reconstructs mammalian phylogenetic relationships remarkably correctly, including internal topologies [20,38,42,43,44], whereas the high mutation rate of the D-loop makes it suitable for analyzing recent evolutionary events and resolving relationships at lower taxonomic levels; however, it is often used alongside other markers since the rapid mutation rate can lead to homoplasy, and the moderately conserved nature of RBP3 makes it easy to compare across different species, which is helpful in studying evolutionary links over long periods. In addition, as a single-copy gene, it simplifies comparisons across species from a common ancestral gene without the complications of gene duplication and loss.

The amplified products were sequenced using the same primers with an ABI sequencer (Applied Biosystems, Waltham, MA, USA), and the chromatograms were assembled in Geneious Prime^®^ 2024.0.5 (www.geneious.com (accessed on 6 July 2024)) with default settings. We sequenced 1003 samples for Cytb (157 *H. endorobae* and 846 *P. jacksoni*), from which we subsampled to sequence the other two genes: 24 D-loop genes and 23 RBP3 *genes* for *H. endorobae* and 50 D-loop genes and 51 RBP3 *genes* for *P. jacksoni*. For a more comprehensive genus-wide analysis, we downloaded all *Hylomyscus* and *Praomys* sequences in GenBank [45], mentioning “Cytb”, “D-loop”, and “RBP3 or IRBP” in the descriptions, and organized them into their respective alignments.

The alignments were processed using MUSCLE [46], after which ‘Gblocks’ [47] was used to remove ambiguously aligned fragments, and ClipKIT [48] was used to analyze parsimony-informative sites, which can be more phylogenetically informative. The final gene datasets were organized into mitochondrial, nuclear, and combined gene datasets after concatenation in ‘Concatenator’ [49]. Subsequent analyses were performed using alignments of individual genes (Cytb, D-loop, and RBP3), combined genes (Cytb + D-loop + RBP3), and mitochondrial genes (Cytb + D-loop).

### 2.4. Data Analysis

To comparatively investigate the distribution and diversity of *P. jacksoni* and *H. endorobae* across forest fragments in Kenya, we analyzed their genetic diversity, phylogenetics, divergence, biogeography, population structure, and niche modeling.

#### 2.4.1. Phylogenetic Analysis and Species Delimitation

To accommodate evolutionary complexity based on the actual nature of our alignments, we reconstructed phylogenetic relationships using maximum likelihood (ML) and Bayesian inference (BI) methods, applying substitution models selected as the best-fit to our datasets in ModelFinder based on the Bayesian inference criterion [50]. The ML analysis was performed using IQ-TREE [51], with branch support estimated from 100,000 ultrafast bootstrap replicates [52]. The BI analysis was performed in BEAST v2.7.7 [53] using two independent runs, each constituting 100 million MCMC chains sampled at 10,000 intervals, with the relaxed log-normal clock priors, which allow for rate variation among branches and the Yule Process speciation model, which assumes a constant speciation rate over time. The BI results were visualized in Tracer to diagnose sampling adequacy (effective sample size values of 200 and higher were taken as adequate sampling), and the log and tree files from the two runs were combined using LogCombiner and annotated using TreeAnnotator [54] using the maximum clade credibility tree, with a 10% burn-in applied.

We performed species delimitation using the ML and BI trees and corresponding alignments using the branch-cutting method; BCUT [55], the multirate Poisson tree processes algorithm; mPTP [56], the single-threshold general mixed Yule coalescent model; GMYC [57], and the assembly of species using automatic partitioning methods; ASAP [58]. We inspected and resolved the differing number of species units suggested by different methods (Supplementary File S3) into a consensus clustering scheme based on morphological characterization, search matches in the BLAST database [59], and distribution accounts in the latest checklists—Musser and Carleton [60], Hutterer [61], Musila et al. [27], Wilson et al. [26], and Wilson et al. [62].

#### 2.4.2. Genetic Diversity and Population Structure Analyses

We used haplotype networks to inspect the genealogical relationships between the delimited species units. The haplotype networks were reconstructed and visualized in PopART v.1.7 [63] using the median-joining network algorithm [64], which visualizes the relationships and frequencies of haplotypes in a population and helps identify population structure [63]. The genetic diversity and divergence within and between species units [65,66] were explored using various genetic diversity indices in DnaSP v 6.12.03 [67] and MEGA11 [68].

#### 2.4.3. Demographic Structure, Lineage Divergence, and Historical Demography Analyses

We analyzed species’ demographic structure, including lineage divergence and phylogeographic and historical demography, using the most complete dataset, Cytb. The input trees were constructed in BEAST using Cytb haplotype alignment with log-normal relaxed clock priors and two 50 million generations sampled every 5000 generations. The time divergence calibrations were constrained using the conventional long-term evolutionary rate for all substitutions in the Cytb gene—0.024 base substitutions/lineages per million years [69]. Each analysis used a random starting tree and the coalescent Bayesian skyline prior. The secondary calibration priors were based on the most recent common ancestor ages (Table A2). The parameter convergence was evaluated in Tracer v1.7.2 [70].

We inferred historical biogeography using ancestral geographic distribution reconstructions of the time-calibrated trees in RASP v.4.4 [71] based on best-fit models selected by BioGeoBEARS [72,73]—the dispersal–extinction–cladogenesis (DEC) model [74]. The DEC model models how species distributions change over time using a species tree (with branch lengths scaled to evolutionary divergence times) and geographical areas where the species (tree tips) occur to describe their geographic range evolution through three key processes: dispersal, extinction, and cladogenesis [73,74]. Different sampling sites were used as ancestral states—Mt. Kenya, Aberdares, Mt. Elgon, Cherangani, Mau, and Loita.

We used Tajima’s D and Fu’s Fs to test for departures from neutral historical demographic processes, supporting population growth, bottlenecks, or selection. A negative Fu’s Fs supports a unimodal mismatch distribution, indicative of population expansion, whereas a positive Fu’s Fs can be associated with a multimodal mismatch distribution, suggesting substructure, bottlenecks, or balancing selection [75]. We used mismatch distributions and Bayesian skyline plots (BSPs) to reconstruct historical demography. The BSP estimates past population dynamics directly from genetic data without predefined demographic models, utilizing coalescent theory within a Bayesian framework to infer changes in effective population size over time, an approach that does not rely on parametric assumptions about population changes. It is particularly useful for studying historical biogeography, population fluctuations, and evolutionary radiations [75]. Relatedly, mismatch analysis can also provide evidence about past events such as expansions, bottlenecks, and the degree of genetic structure within populations [76].

#### 2.4.4. Morphological Trait Analysis

The external measurements recorded in fieldnotes are representative of size-related ecological strategies, including body mass, head–body length, tail length, hindfoot length, and ear length (Data Table S1). We analyzed the multivariate differences in traits across different sampled sites and between sexes using the *adonis2* function from the vegan R package [77], which employs a Euclidean distance matrix—the model included effects for site, sex, and their interaction—*adonis2(trait_data ~ site + sex + site:sex + (1 | as.numeric(siteID)), method = “euclidean”)*.

#### 2.4.5. Climate and Human Activity Impacts on Species Ranges

We employed species distribution modeling and land-use-land-cover change analysis to assess the current and projected habitat suitability for the Kenyan *P. jacksoni* and *H. endorobae* populations. We used 19 bioclimatic variables retrieved from Philipp et al. [78], including monthly temperature and rainfall, annual trends, seasonality, and extreme conditions, which are critical for species survival and serve as standard indicators in ecological studies to evaluate the impacts of climate change on species distributions [79]. We used the GFDL Earth System Model version 4.1 dataset [80] and the “business-as-usual” scenario—the representative concentration pathway 8.5—which represents a scenario with unmitigated climate change and the most significant impacts from increasing greenhouse gas concentrations [81]. The RCP 8.5 model is considered a more practical scenario because of the current trends of continued global reliance on fossil fuels, slow progress in emission reduction, and insufficient political and economic commitment to climate change mitigation [82]. Climate averages from 1981–2010 were used as the baseline, and those from 2011–2040, 2041–2070, and 2071–2100 were used as the future scenarios. The variables were first standardized to the same resolution (approximately 1 km^2^) using the ‘terra’ R package [83].

The species occurrence data and environmental variables were processed using Wallace ([84,85]; Supplementary File S2). Species distribution modeling was implemented in Maxent v3.4.1. We used Maxent v3.4.1 [86] to model species distribution. We divided the data into 75% for model training and 25% for validation to improve the model’s predictive accuracy and generalizability. We conducted ten replicates with jackknife resampling to assess variable importance, and the model was set to run for up to 1000 iterations. We employed the maximum training sensitivity and specificity threshold rule to establish binary predictions. We evaluated the model’s accuracy using the area under the receiver operating characteristic curve, a widely accepted metric for assessing species distribution models. Finally, we classified the modeled habitat suitability into four categories: 0–0.25, 0.25–0.50, 0.50–0.75, and 0.75–1.00, to interpret the magnitude of temporal changes in habitat availability for the species.

We also analyzed projected changes in the area under various land uses and land covers in four future periods (2025, 2050, 2075, and 2100) using four carbon emission scenarios (representative concentration pathways 2.6, 4.5, 6.0, and 8.5). We used the land-use and land-cover change scenarios and classifications from Li et al. [87]. For a more robust understanding of the habitat area change for the two species, we considered two cases: (i) the species’ known range and (ii) just within Kenya (which is relatively comparable to our new field collection sites).

## 3. Results

### 3.1. Sampling/Sample Summary

We recorded 1343 samples from the field surveys that were identified as belonging to the two species; 307 *Hylomyscus endorobae* samples from Mau and Mt. Kenya only, and 1036 *Praomys jacksoni* samples from all the sites (Kakamega, Loita, Mau, and Mt. Kenya). We sequenced 1002 samples for Cytb, which were then used for subsampling to sequence the other two genes: 74 RBP3 sequences—*H. endorobae* (23) and *P. jacksoni* (51), and 73 D-loop sequences—*H. endorobae* (24) and *P. jacksoni* (49). We also downloaded corresponding *P. jacksoni* sequences from GenBank, including 410 Cytb, 162 D-loop*,* 56 RBP3, and 190 Cytb, 32 D-loop, and 23 RBP3 sequences for *H. endorobae.* We obtained 1003 samples across the surveyed sites identified under *H. endorobae* 157 and *P. jacksoni* 846 (Data Table S1). After cleaning the GenBank-downloaded sequences (i.e., removing ambiguous nucleotides, trimming sequences to regions matching the respective genes, and curating species names to be more informative), we retained 2869 across the three genes that were combined with the novel sequences (Data Table S2).

### 3.2. Morphological Differentiation

**Figure 2:**
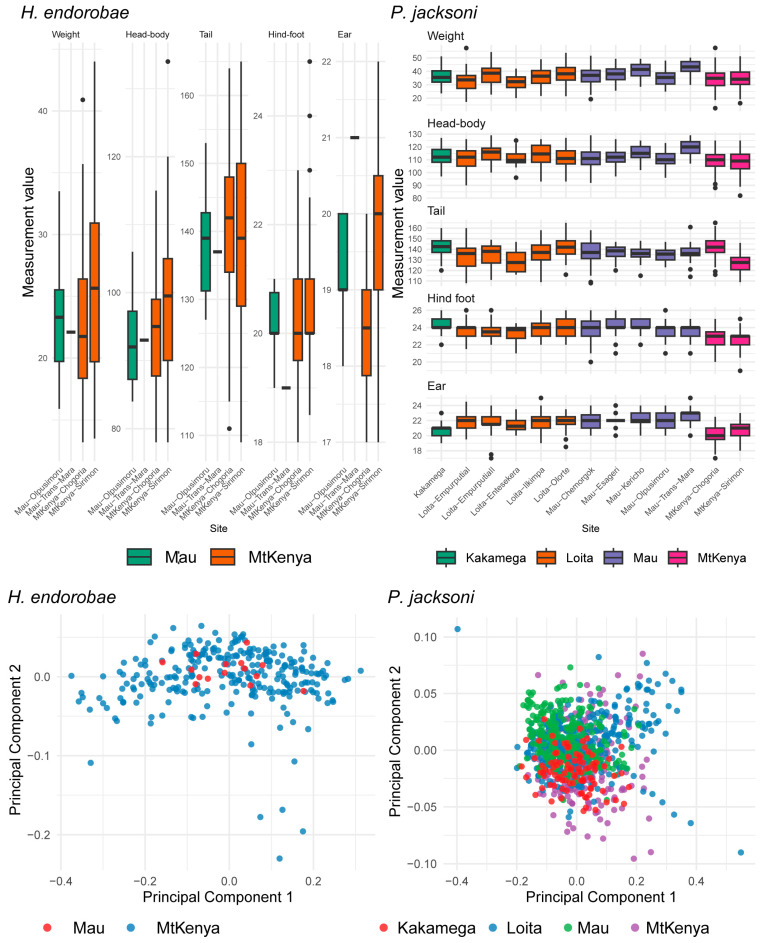
Morphological differentiation of the Kenyan *Praomys jacksoni* and *H. endorobae* samples from different survey sites.

Among the localities, the sampling site had a significant effect on the traits, explaining 1.56% (*p*: 0.185, *F*_3,277_: 1.56) of the variation in *H. endorobae* and 18.32% of the variation in *P. jacksoni* (*p*: < 0.001, *F*_13,886_: 16.298). We also detected a statistically significant effect of sex on the external traits across the sampled sites, with a 4.37% proportion of variation in the traits explained by sex (*p* < 0.001, *F*_2,886_: 25.28) in *P. jacksoni* and 2.95% variation explained in *H. endorobae* (*p*: 0.007, *F*_2,277_: 4.41). The interaction effect between site and sex on the traits was significant, explaining 5.61% of the variation in *H. endorobae* (*p*: 0.002, *F*_3,277_: 5.595) and 3.12% of the variation in *P. jacksoni* (*p* < 0.001, *F*_13,886_: 2.773). However, the PCA plots did not reveal patterned clusters unique to the different sites (Figure 2; Supplementary File S3). Univariate comparisons revealed that the Mt. Kenya Sirimon *H. endorobae* samples were larger than those of Chogoria and Mau for all characters, whereas the Mau samples, on average, had larger bodies than those from Mt. Kenya, Kakamega, and Loita for *P. jacksoni* (Supplementary File S3).

### 3.3. Phylogenetic Associations

**Figure 3:**
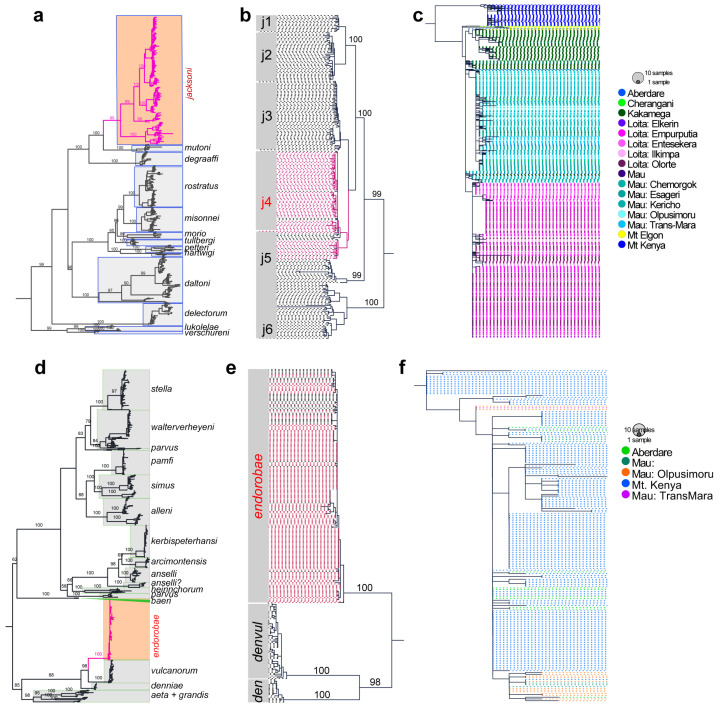
Mitochondrial cytochrome b phylogenies for the *Praomys* and *Hylomyscus* genera. (**a**) Genus *Praomys*, (**b**) *Praomys jacksoni* complex, (**c**) Kenya’s *Praomys jacksoni*, (**d**) genus *Hylomyscus*, (**e**) *Hylomyscus denniae* species group, and (**f**) *Hylomyscus endorobae*.

The Cytb dataset retrieved robustly supported monophyletic clades that matched recognized species (Figure 3). The *Praomys* Cytb tree retrieved well-supported monophyletic clades matching recognized valid species names (Figure 3a), including the subclades (i.e., in Mizerovská et al. [17]) within the *P. jacksoni* complex (Figure 3b). Similarly, the *Hylomyscus* tree was well resolved along recognized species clades (Figure 3d). *H. endorobae was* supported as a monophyletic clade within the *H. denniae* group (Figure 3e). Among the Kenyan samples, *P. jacksoni* presented very distinct geographical effects on the phylogenies, with those from Mt. Kenya being in an easily discriminable monophyletic clade that was sister to a clade containing all the other locality samples (Figure 3c). Other sites also presented distinct clade clustering topologies—Mt. Elgon + Cherangani, Kakamega, Mau, and Loita. On the other hand, the *H. endorobae* tree did not show patterned geographical structuring topologies unique to the sampling sites; however, the Mau samples formed a subclade with the Mt. Kenya + Aberdares samples (Figure 3f). The D-loop and RBP3 trees did not reveal unique topologies, although RBP3 was more phylogenetically uniform, as it did not retrieve systematic associations that could be delineated as unique gene pools, unlike the D-loop trees that reflected the Cytb topology (Supplementary File S4). Thus, the trees from the concatenated mitochondrial (Cytb + D-loop) and combined-genes dataset mirrored the topology of the Cytb tree (Supplementary File S4).

### 3.4. Genetic Diversity and Genetic Relationships

The Cytb between-site genetic divergence in the Kenyan *P. jacksoni* samples ranged from 0.6–2.8%, with the Mt. Kenya samples being the most divergent, with at least 2.44% K2P distance from all other sites (Table 1 and Table 2). However, the within-site divergence distances were notably low overall and were highest in the western Kenya highlands (Mt. Elgon 0.97%, Kakamega 0.62%, Cherangani 0.58%) compared with Mt. Kenya 0.43%, Aberdare 0.32%, Mau 0.32%, and Loita 0.23%. These divergences were largely reflected in the within-site locality groupings, such that none of the Loita Hills or Mau fragments presented uniquely high within- or between-group divergence. At the genus level, the within-group genetic divergence (0.32–5.87% K2P distance) was significantly lower than the between-group genetic divergence (5.7–18.9%). The divergence rates within (3%) and between (11.5–17.7%) the *P. jacksoni* samples and other species were among the highest. In the *H. endorobae* samples, Mt. Kenya was more divergent within (Mt. Kenya 0.301%, Mau 0.195%, Aberdare 0.135%) and between (0.24–0.38%) the sites (Table 1 and Table 2). In the genus *Hylomyscus,* the *denniae* group was one of the least genetically diverse but still presented relatively high genetic distances between the constituent species: 7.1% for *endorobae–dennia,* 7.2% for *endorobae–vulcanorum,* and 6.8% for *denniae–vulcanorum*; genetic divergence was highest in *parvus* (K2P distance: 9.6%) and *simus* (4.4%) samples and lowest within the *kerbispeterhansi*, *endorobae*, *denniae*, and *grandis* samples (0.3%), with between-clade distances ranging from 3.4 to 19.7%—Supplementary File S5.

**Figure 4:**
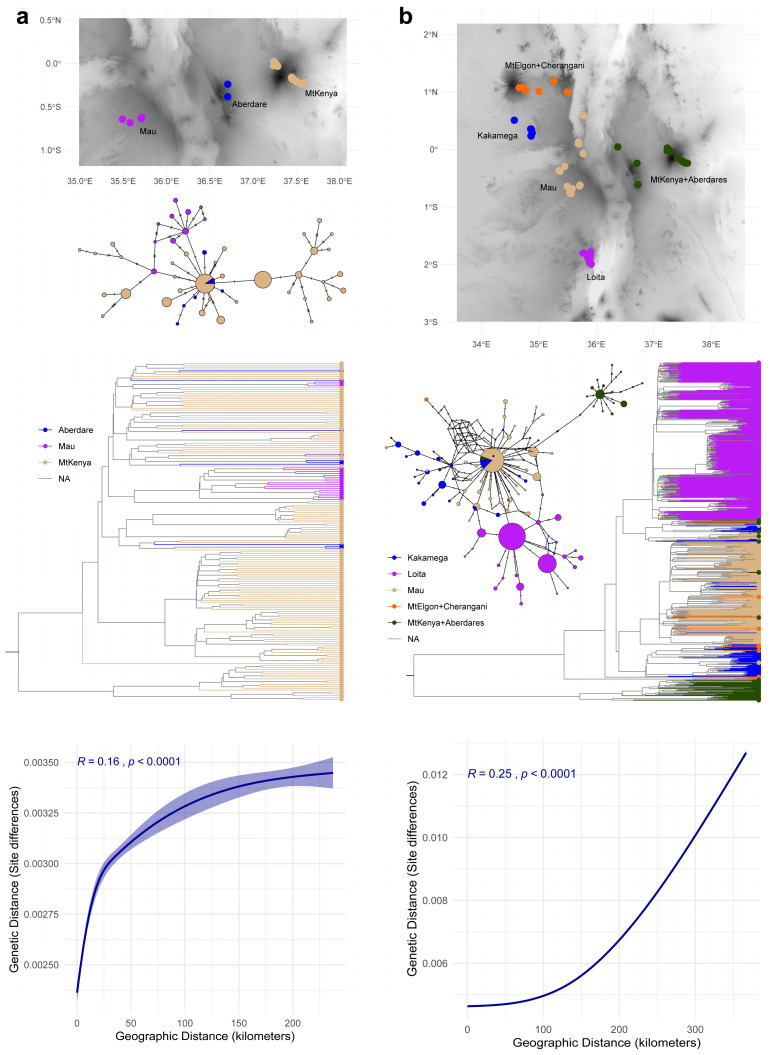
Geographical impacts on genetic structuring within *Hylomyscus endorobae* and *Praomys jacksoni* in Kenya. Panel (**a**) shows the *H. endorobae* results, and panel (**b**) shows the *P. jacksoni* results. The geographical distributions of the samples are overlaid on the elevation layer (darker corresponds to high elevations) in the top figures. The haplotype networks are also shown in corresponding panels, illustrating the genealogical clustering of samples based on localities. The phylogenetic trees in the middle figures are colored based on the sampling locality IDs and match the distribution and haplotype network colors. The correlations between geographic distances (inferred from latitude–longitude sample records) and genetic distances (pairwise nucleotide differences) are shown in the bottom figures.

There were also significant geographical isolation effects on the genetic diversity and clustering scheme with a relatively weak positive correlation between geographic and genetic distance in *H. endorobae* but a more pronounced increase in genetic distance as geographic distance increased in *P. jacksoni*. Despite the associations being statistically significant in both species, only 2.56% and 6.25% of the genetic diversity in *H. endorobae* and *P. jacksoni,* respectively, was attributable to the isolation-by-distance effect (Figure 4).

Haplotype networks largely reflected the pattern of lineage and site relationships in the phylogenetic tree (Figure 4).

### 3.5. Evolutionary Divergence, Historical Biogeography, and Population Dynamics

**Figure 5:**
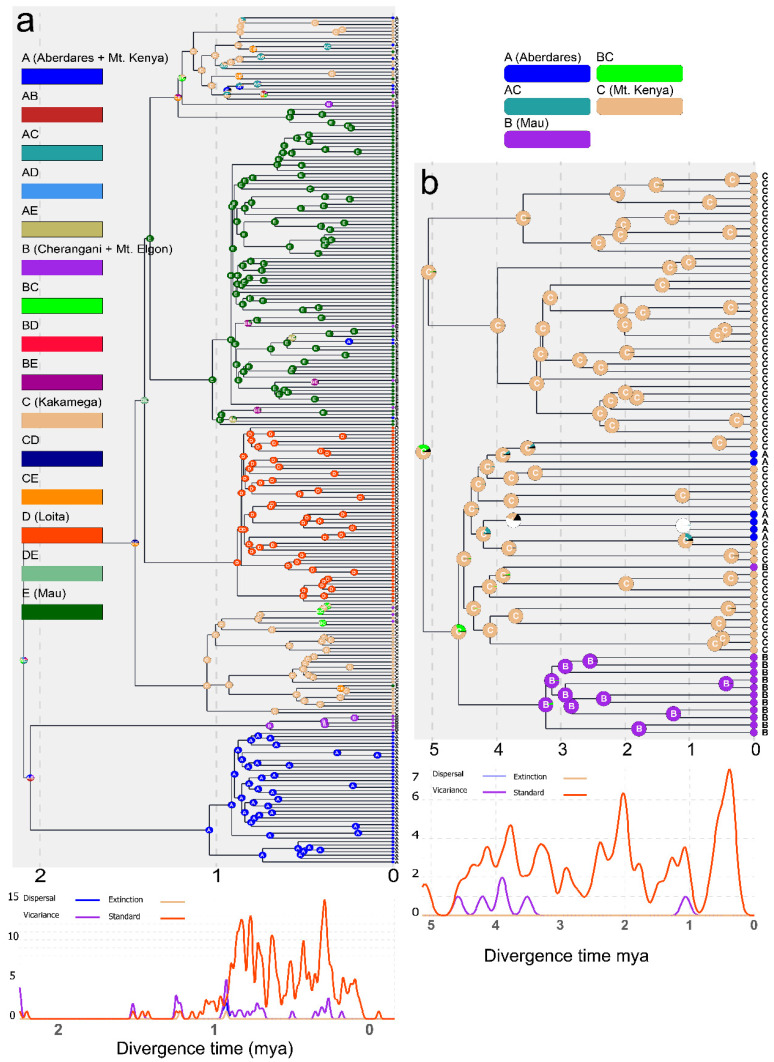
Ancestral area reconstructions of *Praomys jacksoni* (**a**) and *Hylomyscus endorobae* (**b**) based on the dispersal–extinction–cladogenesis model [74] implemented in RASP [71]. The major sampling sites were used as the biogeographical states and are labeled in the legends with matching color schemes.

Divergence time estimates reveal a 2.09 (HPD: 0.96–3.37) ma divergence onset within the Kenyan *P. jacksoni,* which resulted in two major splits: Mt. Kenya + Mt. Elgon versus the rest of the localities (Aberdares, Kakamega, Loita, Mau). The two clades identified (Mt. Kenya and Loita Hills) were monophyletic per sampling locality, i.e., all the samples clustered into single clades, of which the Mt. Kenya clade split from Mt. Elgon clade 2.05 ma (HPD: 0.93–3.35) was the oldest, followed by the split of Kakamega + Cherangani + Mt. Elgon clade 1.46 (HPD: 0.53–2.62) and a pure Loita Hills clade split 1.41 ma (HPD: 0.49–2.54) from a clade comprising mostly Mau samples and Aberdares, Kakamega, and Cherangani samples. For *H. endorobae*, a split of 5.14 ma (2.99–7.3) separated Mt. Kenya and Mt. Kenya + Aberdares + Mau clade, with the monophyletic Mau clade splitting 4.59 ma (2.31–7.02) (Figure 5).

Historical biogeography analyses reveal that divergences between the main clades (corresponding to the sampling sites) were largely unresolved, with most not being species-level splits but rather radiating populations, probably due to suitable ecological conditions. For *H. endorobae*, the ancestral range was predicted to have been in Mt. Kenya, with the splits within the respective Mt. Kenya or Mau lineages attributed to dispersals within these ecosystems (Figure 5). In *H. endorobae,* standard events defined radiations from ancestral states, with prominent peaks at 3.8, 2 ma, and near the present time. There were also vicariance events, which appeared relatively low and constant throughout the timeline compared with the standard, with minor peaks at approximately 4.5–3.5 and 1 ma, suggesting that periods of geographic or ecological separation influenced species divergence. There were no dispersal or extinction events.

On the other hand, for *P. jacksoni,* the source ancestral states of the Kenyan populations were not resolved, with no credible assignment of ancestral forest sources for the main clade divergences (Figure 5). Divergences within the clades were estimated to have originated within the corresponding sites, i.e., following dispersal, major lineages within clades arose within the same areas.

**Figure 6:**
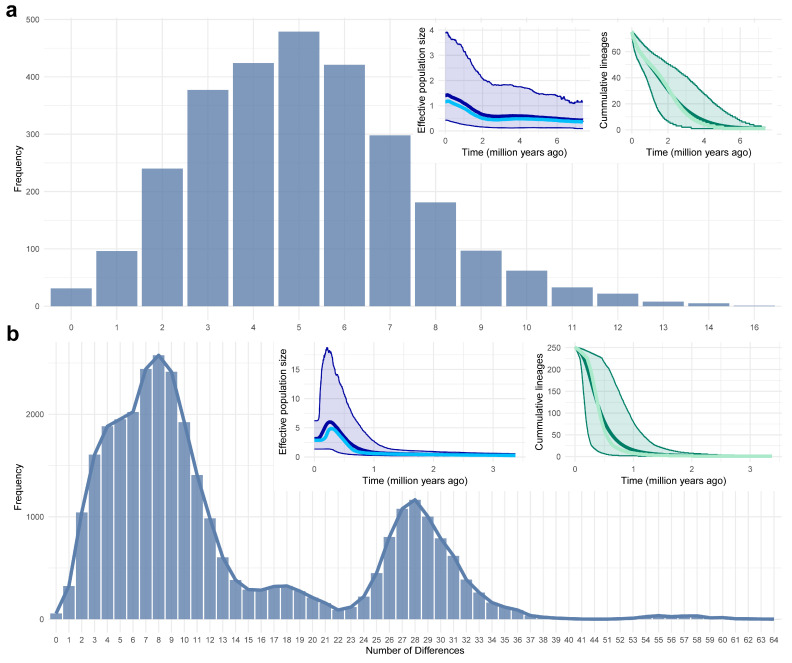
Population dynamics analysis of *Hylomyscus endorobae* (**a**) and *Praomys jacksoni* (**b**) in Kenya. The main figures (bar plots) show the mismatch distribution analysis, with the *y*-axis showing the frequency of pairwise nucleotide differences between sequences. The inset plots show the Bayesian skyline plots of population change (*y*-axis) over evolutionary time (*x*-axis) to the left and corresponding lineages through time to the right.

The population dynamics analyses reveal notable population increases for both *H. endorobae* and *P. jacksoni* (Figure 6). *H. endorobae* exhibited a bell-shaped mismatch analysis plot with a unimodal distribution with a peak at approximately five different locations, suggesting low nucleotide differentiation between sequences, with very few sequences exhibiting many differences (>10). On the other hand, *P. jacksoni* showed a bimodal mismatch distribution with two prominent peaks; the first, approximately 6–7 differences, was larger, reflecting close relationships between most sequences, whereas the second, approximately 28–29 differences, represented another group of sequences with a higher level of divergence between sequences. There was also a long tail extending beyond 40 differences but with a lower frequency.

### 3.6. Climate- and Human-Driven Habitat Changes

**Figure 7:**
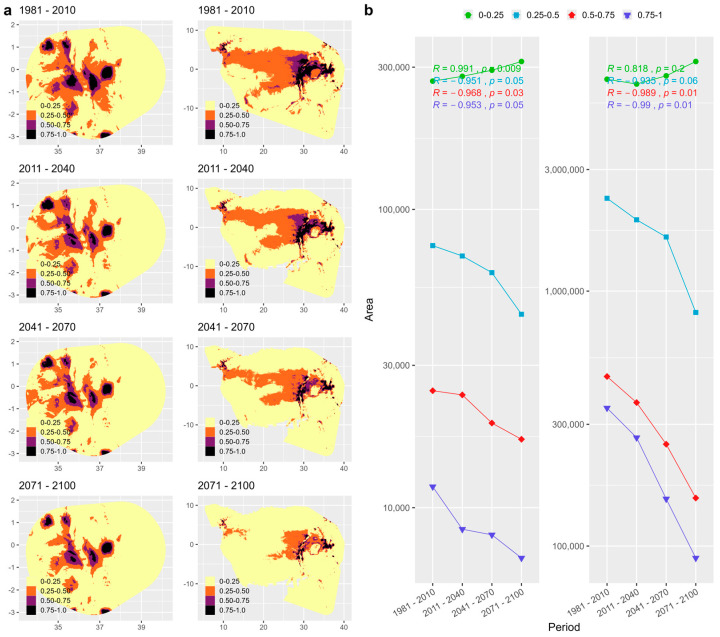
Habitat suitability maps and summary graphs of species distribution modeling projections for habitat suitability scenarios for *Praomys jacksoni* and *Hylomyscus endorobae*; (**a**) shows the range-wide changes to the modeled habitat suitability classes, with (**b**) showing the corresponding quantitative changes summarized into periods by area change associations. In both (**a**,**b**), the panels to the left represent *H. endorobae*, whereas those to the right represent *P. jacksoni*. In (**a**), the x axes represent longitude and the y axes represent latitude.

**Figure 8:**
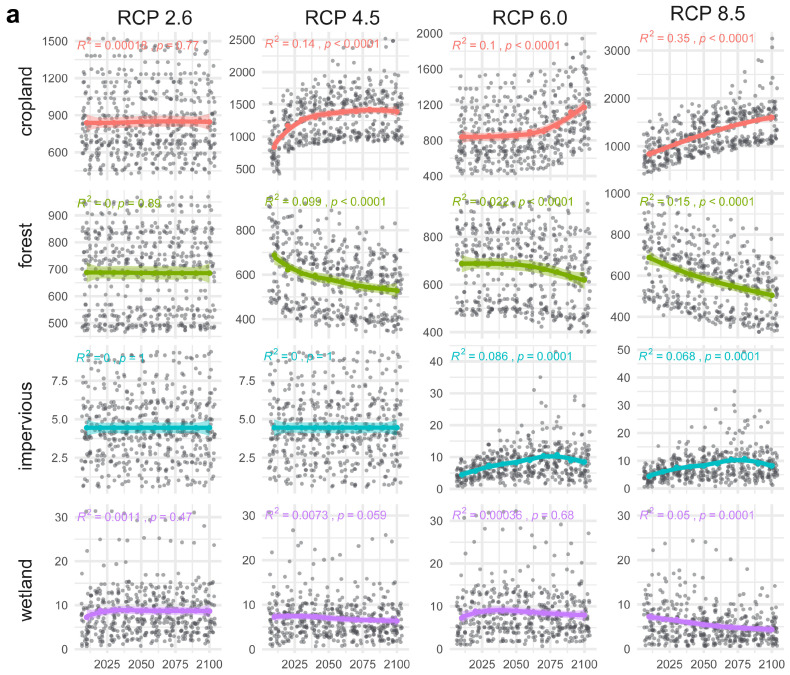

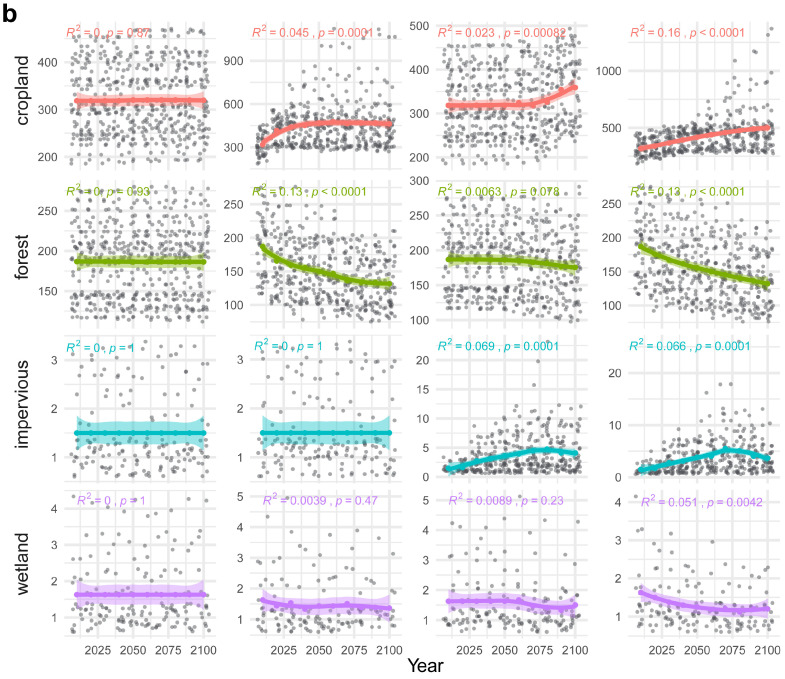
Projected land-use changes within the IUCN-recorded species distribution range (**a**) and within the species’ known distribution range in Kenya (**b**) for *Praomys jacksoni* and *Hylomyscus endorobae*. The range of *H. endorobae* is entirely nested within the *P. jacksoni* range (see Figure 1).

The SDMs consistently lost the modeled suitable habitats under the future climate change scenarios. For both species, the SDMs revealed that suitable areas mainly overlapped with areas where the species have been recorded. For *H. endorobae*, the highly suitable areas were in the Mt. Kenya, Cherangani, Mau-Aberdares, and Mt. Elgon regions, with an isolated patch in the portion of Mt. Kilimanjaro that remained in the raster cutout (Figure 7a). The overall habitat area change was negative for all >0.25 suitability classes, with a steady decline until the 2100s, whereas the low suitability (<0.25) areas steadily increased during this period. The *P. jacksoni* moderate to highly suitable ranges will progressively reduce to highly restricted areas in the Albertine Rift and East African mountains, shrinking in all moderate to highly suitable areas (>0.25), with only expansion in the low-suitability areas, 0.00–0.25 (Figure 7a). While the central African region was modeled to be not the most suitable for this species (most of it fell under the 0.25–0.50 class), these moderately suitable habitats will also be progressively lost, nearly disappearing by the 2100s (Figure 7b). The land-use–land-cover change analyses under the same RCP mirrored the SDM changes, indicating steady forest declines and cropland increases in the species’ respective ranges and in Kenya (Figure 8). Human infrastructure land uses such as impervious areas or land covers vulnerable to pressure from human activity are also predicted to increase and decline, respectively.

## 4. Discussion

*Contrasting divergence and population trends between Praomys jacksoni and Hylomyscus endorobae:* The recovered phylogenetic relationships predominantly corroborate established and recent research advances of these species and further elucidate several novel insights into habitat-level genetic and morphological affinities between habitats/populations. For more detailed genera and species-level taxonomic insights, please refer to the excellent recent publications on *Hylomyscus*—[18,28,88] and the *Praomys jacksoni* complex—[15,16,17,89], and the well-curated and updated mammal diversity database [23]. In our study, *H. endorobae* formed a monophyletic cluster without any discernible systematic differentiation between sites. In contrast, *P. jacksoni* exhibited site-level clustering, indicating a stronger correlation between genetic distance and geographical distance. These results suggest that the two species have experienced unique evolutionary histories, underlain by the interaction of influences from orographic events, human activity, and paleoclimatic influences [90]. These genetic variation patterns could also stem from differences in ecological strategies where *P. jacksoni* could have effectively adapted ecologically to local conditions due to stronger biotic filtering, gaining more genetic distinction specific to these conditions in the process. The factors driving these fast ecological fitness adaptations can range from competitive advantages in resource extraction driven by diet, activity, and character partitioning. On the other hand, *H. endorobae* seem to have faced minimal ecological pressure that required high specialization to favor survival, probably due to low levels of competition [91]. The observed genetic divergence can also be attributed to distinct ecological and behavioral traits, underscoring species-specific responses to habitat fragmentation and environmental pressures, influencing their respective genetic patterns. To date, *P. jacksoni* has been recorded from a variety of forested environments across its range, which is restricted to Sub-Sahara Africa, including lowland and montane forests, and is more adaptable to different forest types, spanning both primary and secondary forests [15,16,17]. Its distribution underscores an adaptability, which can facilitate gene flow across fragmented landscapes, leading to greater genetic diversity and regional differentiation. In contrast, *H. endorobae* is a stricter forest specialist, having been recorded only from the wet montane forests at high elevations in the Kenyan Rift mountains [18,28,88]. This restricted habitat preference and potentially lower dispersal capacity result in a more homogeneous genetic structure across its range.

We did not find support for the hypothesis that older evolutionary lineages, such as *H. endorobae*, exhibit greater genetic diversity than relatively younger lineages, such as *P. jacksoni*, since an extended evolutionary timeline contributes to richer genetic complexity because more mutations, adaptations, and survival mechanisms developed across varied environmental conditions [92,93,94]. Instead, we found surprisingly low genetic diversity in *H. endorobae* compared with *P. jacksoni*, suggesting that a complex interplay of evolutionary processes beyond the mere time of divergence and other processes such as population bottlenecks, and limited gene flow, selective pressures [95], might have played a more significant role in shaping the genetic landscapes of these populations. Moreover, the *H. endorobae* population analysis retrieved unimodal, bell-shaped mismatch analysis topology, consistent with a recent population expansion, with the Bayesian skyline and lineage-through-time plots also supporting scenarios of recent population expansion ca. 2.4 ma (early Pleistocene). For *P. jacksoni*, the bimodal mismatch corresponds with the two clades retrieved from the phylogenetic trees and haplotype networks, reflecting a recently expanding population or relatively homogeneous population [75,76]. The second peak could be a separate subpopulation or a historical event that caused a split or divergence within the main population, while the long tail corresponds to a highly divergent clade from the Mt. Kenya samples. While these results are based on geographic isolation from the other samples, comparisons with the *H. endorobae* patterns raise some critical biogeographic questions. Several contending scenarios can apply to these diversity and divergence patterns, including recent population expansion, divergence over a more extended period, rapid expansion following a bottleneck, or founder effect divergence [96,97]. The contending scenario of a hybridization or admixture event between two previously isolated populations is much more unlikely due to the pan-African nature of the *P. jacksoni* lineage; the Mt. Kenya population is unlikely to be the “core” population, and the second peak could also represent individuals with mixed ancestry, such as in cases of reticulate population interactions [19,98]. The contrasting population trends observed in *P. jacksoni* and *H. endorobae* suggest that, among the scenarios of vicariance, dispersal, or in situ divergence, in situ divergence is the most plausible model for divergence and radiation in the Kenya highlands. The genetic structure of *P. jacksoni* indicates limited gene flow between the eastern and western highlands of the East African Rift, but with more recent divergence time estimates, which can be explained by in situ speciation, where new gene pools, which may eventually develop into distinct species, evolve within the same geographic region without physical barriers to gene flow [5,16,90,96,99]. The scarcity of dispersal events and almost no instances of vicariance or extinction in the ancestral state reconstructions further support this concept.

*Discordant tree topologies between genes:* We investigated discrepancies among phylogenetic topologies derived from different genes, focusing on the CYTB gene, which provided a robust basis for species delineation and aligned well with the existing taxonomic literature. In contrast, the gene trees based on the D-loop and RBP3 genes exhibited significant discrepancies and had lower phylogenetic informativeness. These inconsistencies may be explained by factors such as incomplete lineage sorting and insufficient sampling, both of which can impact the resolution of phylogenetic trees [100]. Furthermore, our findings are relevant to the ongoing debate about the optimal balance between taxon sampling and gene (character) sampling in phylogenetic studies, as researchers continue to explore strategies to increase phylogenetic resolution [100].

*Geographical impacts and historical biogeography hypotheses: Hylomyscus endorobae* portrayed an asymptotic genetic distance relationship with geographic distance, unlike the exponential increase in *P. jacksoni*. Theoretically, populations occurring close to each other are more alike, linked by larger amounts of gene exchange, than they are with more distant populations. As such, the probability of affecting gene flow by dispersion decreases with the distance between the source population and the recipient (or sink) population. For *P. jacksoni,* migrant species from the broader westward range could act as a source population for any declines in the Kenyan range [99,101,102,103,104,105]. The striking separation between Mt. Kenya *P. jacksoni* and the other sites cannot be explained by significant vicariance-related dynamics in the region, such as the Oligocene Rift Valley Formation, which predates the observed divergence times [106,107]. We hypothesize that populations on either side have accumulated genetic diversity/divergence in relative isolation following initial Plio-Pleistocene *Praomys* and *Hylomyscus* colonization of these highlands [15,16,17,18,28,88,89,108,109,110]. Over time, habitat-fragmenting human activities and geographical separation accentuated these genetic divergences. For both *H. endorobae* and *P. jacksoni*, our results cannot reliably support contemporary forest fragmentation as a key driver of the observed population structuring (i.e., acting as an effective gene flow barrier). However, because fragmentation severs population interaction links as distances between forests become increasingly unsuitable as stepping stones for these species [111], further forest declines will impose stronger gene flow barriers, especially for *P. jacksoni,* and likely onset incipience for different well-defined populations, such as Mt. Kenya + Aberdare Ranges, the Loita Hills, Kakamega Forest, and Mt. Elgon + Cherangani Hills.

*Climate change impacts on species habitats:* Our species distribution modeling and analysis of land-use and land-cover changes suggest that future suitable range contractions for both species will be exacerbated by increased human-dominated landscapes such as croplands and settlements. While *H. endorobae* is endemic to the Kenya highlands, *P. jacksoni* has a much larger range outside Kenya. Thus, the modeled contractions in suitable habitats for *H. endorobae* will have greater effects on its genetic diversity and population. The genetic pool of the Kenyan *P. jacksoni* is likely rejuvenated from the extended range [112].

*Implications for biodiversity conservation*: The genetic structuring and diversity patterns observed between *P. jacksoni* and *H. endorobae* have significant implications for biodiversity in Kenya’s highland forests. The greater genetic diversity and biogeographic structuring seen in *P. jacksoni* suggest that forest fragmentation may be driving localized adaptations, with isolated populations potentially experiencing distinct selective pressures. In contrast, *H. endorobae’*s lack of geographic structuring could indicate a broader resilience or adaptability across fragmented habitats. These species-specific responses to habitat fragmentation highlight the need for conservation strategies that consider the ecological and evolutionary complexities of different species and habitat connectivity between remaining forest fragments. Expanding sampling efforts across Kenya’s highland forests is also crucial to achieving a more comprehensive understanding of the genetic impacts of fragmentation on biodiversity. These endeavors will facilitate more effective conservation actions that preserve diverse adaptive potentials, ultimately helping to maintain the ecological integrity of these forests amid ongoing habitat changes.

## 5. Conclusions

We investigated the evolutionary dynamics and ecological adaptations of *Hylomyscus endorobae* and *Praomys jacksoni* in Kenya’s forests, aiming to enhance our understanding of their phylogenetic relationships, evolutionary trajectories, and vulnerability to climate change within fragmented habitats. The phylogenetic findings align with established and recent insights into lineage divergences, revealing patterned clustering schemes across sites for *P. jacksoni* but not for *H. endorobae*. However, these phylogenetic patterns do not coincide with morphological differences. Furthermore, the genetic variation in *P. jacksoni* increases exponentially with geographical distance, indicating a strong positive correlation between geographic and genetic distances, whereas differentiation within *H. endorobae* asymptotically increases with geographic distance. The major phylogenetic divergences occurred during the Pliocene–Pleistocene transition, with *H. endorobae* exhibiting an older divergence than *P. jacksoni*. Recent population expansions in both species occurred during distinct climate transition periods, with *P. jacksoni* experiencing reticulate or punctuated expansion. Under climate change, suitable ranges for both species will contract, attributed to declines in areas under forest cover and an expansion of human-dominated landscapes, such as croplands. Future studies could elucidate the patterns and processes of ecological and anthropogenic barriers obstructing gene flow across these landscapes with finer genetic and field sampling. Such expanded sampling will also be vital to refining the taxonomic limits of these species within their genera placements and more local gene pools statuses. Moreover, detailed investigations into whether Mount Kenya is a regional’ species pump’ or ‘refugia’ would bridge some critical gaps in explaining the observed patterns of genetic diversity, genealogical relationships, and role in the demographic pathways of older and newer lineages in the region [101].

## Figures and Tables

**Table 1:** The number of base substitutions per site averaged over all sequence pairs within each study site (‘within’ column) and between study sites (displayed in matrices), inferred using the Kimura two-parameter model in MEGA11 [68] using the Cytb dataset. Part (a) shows results for *Hylomyscus endorobae*, and part (b) shows results for *Praomys jacksoni*.

(a) *H. endorobae*								
Site	Within	Mt. Kenya	Mau	Aberdare				
Mt. Kenya	0.301%							
Mau	0.235%	0.358%						
Aberdare	0.135%	0.248%	0.262%					
(b) Kenyan *P. jacksoni*								
Site	Within	Aberdare	Kakamega	Mau	Loita	Mt. Elgon	Cherangani	Mt. Kenya
Aberdare	0.316%							
Kakamega	0.622%	0.607%						
Mau	0.315%	0.427%	0.763%					
Loita	0.233%	0.517%	0.747%	0.700%				
Mt. Elgon	0.975%	1.110%	1.211%	1.307%	1.231%			
Cherangani	0.576%	0.440%	0.715%	0.481%	0.679%	1.206%		
Mt. Kenya	0.430%	2.443%	2.652%	2.664%	2.624%	2.795%	2.579%	

**Table 2:** Genetic diversity within and between *Hylomyscus endorobae* and Kenyan *Praomys jacksoni* based on the Cytb dataset, inferred using DnaSP6 [67].

Statistic	*H. endorobae*				*P. jacksoni*			
Diversity Index	All	Aberdares	Mau	Mt. Kenya	All	Aberdares	Mau	Mt. Kenya
Sequences	181	10	21	150	891	9	287	55
Total sites *	1094	1120	1120	1094	519	519	1113	743
Polymorphic sites	62	6	14	50	95	4	106	37
Mutations	63	6	14	51	100	4	110	38
Haplotypes	49	6	10	34	115	4	87	19
Haplotype diversity	0.908	0.844	0.919	0.881	0.887	0.583	0.941	0.81
Nucleotide diversity	0.003	0.001	0.002	0.003	0.006	0.002	0.003	0.004
Nucleotide differences	3.225	1.511	2.638	3.144	3.24	0.889	3.534	2.801
Fu and Li’s D test statistic	−2.224	−0.939	0.0316	−2.432	−3.257	−1.8	−4.107	−3.54
Statistical significance	*p* > 0.05	*p* > 0.10	*p* > 0.10	*p* < 0.05	*p* < 0.05	*p* > 0.05	*p* < 0.02	*p* < 0.02
Fu and Li’s F test statistic	−2.631	−1.122	−0.372	−2.702	−3.196	−1.948	−3.926	−3.646
Statistical significance	*p* < 0.05	*p* > 0.10	*p* > 0.10	*p* < 0.05	*p* < 0.02	*p* > 0.05	*p* < 0.02	*p* < 0.02
Tajima’s D	−2.147	−1.189	−1.165	−2.003	−2.136	−1.61	−2.419	−2.218
Statistical significance	*p* < 0.01	*p* > 0.10	*p* > 0.10	*p* < 0.05	*p* < 0.01	*p* > 0.05	*p* < 0.0	*p* < 0.01

* Total number of sites (excluding sites with gaps/missing data).

## Data Availability

All original data presented in the study are available in Zenodo (https://doi.org/10.5281/zenodo.13981879) or otherwise available at the respective sources/citations.

## Supplementary Materials

The following supporting information can be downloaded at https://www.mdpi.com/article/10.3390/life14111469/s1: Data Tables S1 (list of field samples) and S2 (list of GenBank samples) and Supplementary Files S1 (extended study areas’ descriptions), References [113,114,115,116,117,118,119,120,121,122] are cited in the supplementary materials, S2 species distribution modeling data processing), References [123,124,125,126,127,128,129,130,131] are cited in the supplementary materials, S3 (species morphological characterization), S4 (phylogenetic trees), S5 (genus-level genetic distances between species units).

## Appendix A

**Table A1:** List of genes amplified with the corresponding primers and PCR settings. *F* = forward primer, *R* = reverse primer. The thermal profile for RBP3 amplification consisted of a touch-up annealing protocol from 52 °C to 56 °C.

Gene	Primers				PCR Conditions
	Primer ID		Primer Sequence	Reference	Denaturation | Annealing | Extension | # Cycles
Cytb	F	14724-L	GGACTTATGACATGAAAAATCATCGTTG	[132]	30 s 95.0 °C | 30 s, 59.0 °C | 60 s, 72.0 °C | 40
	R	14139-H	GATTCCCCATTTCTGGTTTACAAGAC		
D-loop	F	Dloop-L	CTGGTCTTGTAAACCAGAAATG	[14]	30 s, 95.0 °C | 30 s, 56.0 °C | 30 s, 72.0 °C | 35
	R	Dloop-H	CCATCTAAGCATTTTCAGTGCTTT		
RBP3-1	F	RBP-L1	ATGACGAGAGAATGGGCCC	[133]	20 s, 94.0 °C | 20 s, 52.0 °C | 80 s, 72.0 °C | 10
	R	RBP-H1	CTGGTGAGGACCACCACATCCTT		
RBP3-2	F	RBP-L2	ATGGCCAAGGTCCTCTTGGATAACTACTGCTT		20 s, 94.0 °C | 20 s, 56.0 °C | 80 s, 72.0 °C | 20
	R	RBP-H2	CGCAGGTCCATGATGAGGTGCTCCGTGTCCTG		

**Table A2:** The time-calibration parameters used to reconstruct the final phylogenetic tree for estimating phylogenetic diversity indices. The analysis was implemented in BEAST using log normal priors, with ‘mean in real space’ activated and offset set to 0. M = mean, S = standard deviation.

Taxon	Mean	2.5% Quantile	97.5% Quantile	M	S	Estimated Time	Range
*Acomys*	5.76	5.34	6.22	1.751	0.039	5.76	5.30–6.22
*Praomys*	5.69	5.16	6.25	1.737	0.049	5.68	0.00–6.25
*Hylomyscus*	6.56	2.45	12.5	1.780	0.451	5.93	0.00–14.34
*Praomys jacksoni*	2.29	1.24	3.55	0.788	0.292	3.08	2.19–3.90
*Hylomyscus endorobae*	5.53	3.53	8.24	1.686	0.216	5.40	4.17–8.25

## References

[1] Fahrig L. (2003). Effects of Habitat Fragmentation on Biodiversity. Annu. Rev. Ecol. Evol. Syst..

[2] Schlaepfer D.R., Braschler B., Rusterholz H.-P., Baur B. (2018). Genetic effects of anthropogenic habitat fragmentation on remnant animal and plant populations: A meta-analysis. Ecosphere.

[3] Kuipers K.J.J., Hilbers J.P., Garcia-Ulloa J., Graae B.J., May R., Verones F., Huijbregts M.A.J., Schipper A.M. (2021). Habitat fragmentation amplifies threats from habitat loss to mammal diversity across the world’s terrestrial ecoregions. One Earth.

[4] Wilson M.C., Chen X.-Y., Corlett R.T., Didham R.K., Ding P., Holt R.D., Holyoak M., Hu G., Hughes A.C., Jiang L. (2016). Habitat fragmentation and biodiversity conservation: Key findings and future challenges. Landsc. Ecol..

[5] Lopez S., Rousset F., Shaw F.H., Shaw R.G., Ronce O. (2009). Joint Effects of Inbreeding and Local Adaptation on the Evolution of Genetic Load after Fragmentation. Conserv. Biol..

[6] Laland K.N., Odling-Smee F.J., Feldman M.W. (1999). Evolutionary consequences of niche construction and their implications for ecology. Proc. Natl. Acad. Sci. USA.

[7] Males J., Neate-Clegg M.H.C., Tingley M.W. (2023). Building a mechanistic understanding of climate-driven elevational shifts in birds. PLoS Clim..

[8] Maslin M.A., Brierley C.M., Milner A.M., Shultz S., Trauth M.H., Wilson K.E. (2014). East African climate pulses and early human evolution. Quat. Sci. Rev..

[9] Peyron O., Jolly D., Bonnefille R., Vincens A., Guiot J. (2017). Climate of East Africa 6000 14C Yr B.P. as Inferred from Pollen Data. Quat. Res..

[10] Peter B.d. (2004). African climate change and faunal evolution during the Pliocene–Pleistocene. Earth Planet. Sci. Lett..

[11] Maslin M.A., Christensen B. (2007). Tectonics, orbital forcing, global climate change, and human evolution in Africa: Introduction to the African paleoclimate special volume. J. Hum. Evol..

[12] Baxter A.J., Verschuren D., Peterse F., Miralles D.G., Martin-Jones C.M., Maitituerdi A., Van der Meeren T., Van Daele M., Lane C.S., Haug G.H. (2023). Reversed Holocene temperature–moisture relationship in the Horn of Africa. Nature.

[13] Marchant R. (2021). Climate Change in Eastern Africa. Oxf. Res. Encycl. Afr. Hist..

[14] Huhndorf M.H., Kerbis Peterhans J.C., Loew S.S. (2007). Comparative phylogeography of three endemic rodents from the Albertine Rift, east central Africa. Mol. Ecol..

[15] Nicolas V., Missoup A.D., Denys C., Kerbis Peterhans J., Katuala P., Couloux A., Colyn M. (2011). The roles of rivers and Pleistocene refugia in shaping genetic diversity in Praomys misonnei in tropical Africa. J. Biogeogr..

[16] Bryja J., Mikula O., Patzenhauerová H., Oguge N.O., Šumbera R., Verheyen E., Riddle B. (2014). The role of dispersal and vicariance in the Pleistocene history of an East African mountain rodent, Praomys delectorum. J. Biogeogr..

[17] Mizerovská D., Nicolas V., Demos T.C., Akaibe D., Colyn M., Denys C., Kaleme P.K., Katuala P., Kennis J., Kerbis Peterhans J.C. (2019). Genetic variation of the most abundant forest-dwelling rodents in Central Africa (Praomys jacksoni complex): Evidence for Pleistocene refugia in both montane and lowland forests. J. Biogeogr..

[18] Nicolas V., Fabre P.H., Bryja J., Denys C., Verheyen E., Missoup A.D., Olayemi A., Katuala P., Dudu A., Colyn M. (2020). The phylogeny of the African wood mice (Muridae, Hylomyscus) based on complete mitochondrial genomes and five nuclear genes reveals their evolutionary history and undescribed diversity. Mol. Phylogenet. Evol..

[19] Komarova V.A., Kostin D.S., Bryja J., Mikula O., Bryjová A., Čížková D., Šumbera R., Meheretu Y., Lavrenchenko L.A. (2021). Complex reticulate evolution of speckled brush-furred rats (Lophuromys) in the Ethiopian centre of endemism. Mol. Ecol..

[20] Onditi K.O., Demos T.C., Kerbis Peterhans J., Chen Z.Z., Bryja J., Lavrenchenko L.A., Musila S., Verheyen E., Van de Perre F., Akaibe B.D. (2021). Historical biogeography, systematics, and integrative taxonomy of the non-Ethiopian speckled pelage brush-furred rats (Lophuromys flavopunctatus group). BMC Ecol. Evol..

[21] Burton A.C., Beirne C., Gaynor K.M., Sun C., Granados A., Allen M.L., Alston J.M., Alvarenga G.C., Calderón F.S.Á., Amir Z. (2024). Mammal responses to global changes in human activity vary by trophic group and landscape. Nat. Ecol. Evol..

[22] Rowan J., Du A., Lundgren E.J., Faith J.T., Beaudrot L., Campisano C.J., Joordens J.C., Lazagabaster I.A., Locke E.M., Smail I.E. (2024). Long-term biotic homogenization in the East African Rift System over the last 6 million years of hominin evolution. Nat. Ecol. Evol..

[23] Mammal Diversity Database Mammal Diversity Database (Version 1.13) [Data Set]. Zenodo..

[24] Monadjem A., Taylor P.J., Denys C., Cotterill F.P.D., Monadjem A., Taylor P.J., Denys C., Cotterill F.P.D. (2015). Rodents of Sub-Saharan Africa: A Biogeographic and Taxonomic Synthesis.

[25] Burgin C.J., Wilson D.E., Mittermeier R.A., Rylands A.B., Lacher T.E., Sechrest W. (2020). Illustrated Checklist of the Mammals of the World.

[26] Wilson D.E., Thomas E Lacher J., Mittermeier R.A., François T.L. (2019). Volume 7: Rodents II. Handbook of the Mammals of the World (HMW).

[27] Musila S., Monadjem A., Webala P.W., Patterson B.D., Hutterer R., De Jong Y.A., Butynski T.M., Mwangi G., Chen Z.Z., Jiang X.L. (2019). An annotated checklist of mammals of Kenya. Zool. Res..

[28] Kerbis Peterhans J.C., Hutterer R., Krasova J., Doty J., Malekani J., Moyer D., Bryja J., Banasiak R., Demos T. (2020). Four new species of the Hylomyscus anselli group (Mammalia: Rodentia: Muridae) from the Democratic Republic of Congo and Tanzania. Bonn. Zool. Bull..

[29] Mahaney W. (1988). Holocene glaciations and paleoclimate of mount Kenya and other East African mountains. Quat. Sci. Rev..

[30] Mahaney W.C., Harmsen R., Spence J.R. (1991). Glacial-interglacial cycles and development of the Afroalpine ecosystem on East African Mountains: I. Glacial and postglacial geological record and paleoclimate of Mount Kenya. J. Afr. Earth Sci. (Middle East).

[31] Michael C. (2006). Climate Change Impacts on East Africa: A Review of the Scientific Literature.

[32] Jaramillo J., Muchugu E., Vega F.E., Davis A., Borgemeister C., Chabi-Olaye A. (2011). Some like it hot: The influence and implications of climate change on coffee berry borer (Hypothenemus hampei) and coffee production in East Africa. PLoS ONE.

[33] Schüler L., Hemp A., Zech W., Behling H. (2012). Vegetation, climate and fire-dynamics in East Africa inferred from the Maundi crater pollen record from Mt Kilimanjaro during the last glacial–interglacial cycle. Quat. Sci. Rev..

[34] Leclerc C., Mwongera C., Camberlin P., Moron V. (2014). Cropping System Dynamics, Climate Variability, and Seed Losses among East African Smallholder Farmers: A Retrospective Survey. Weather Clim. Soc..

[35] Menegon M., Loader S.P., Marsden S.J., Branch W.R., Davenport T.R., Ursenbacher S. (2014). The genus Atheris (Serpentes: Viperidae) in East Africa: Phylogeny and the role of rifting and climate in shaping the current pattern of species diversity. Mol. Phylogenet. Evol..

[36] Liu X., Rendle-Bühring R., Henrich R. (2018). High-and low-latitude forcing of the East African climate since the LGM: Inferred from the elemental composition of marine sediments off Tanzania. Quat. Sci. Rev..

[37] Musila S., Chen Z.Z., Li Q., Yego R., Zhang B., Onditi K., Muthoni I., He S.W., Omondi S., Mathenge J. (2019). Diversity and distribution patterns of non-volant small mammals along different elevation gradients on Mt. Kenya, Kenya. Zool. Res..

[38] Onditi K.O., Peterhans J.K., Demos T.C., Musila S., Chen Z.Z., Jiang X.L. (2020). Morphological and genetic characterization of Mount Kenya brush-furred rats (Peters 1874); relevance to taxonomy and ecology. Mammal Res..

[39] Sikes R.S., The Animal Care Use Committee of the American Society of Mammalogists (2016). 2016 Guidelines of the American Society of Mammalogists for the use of wild mammals in research and education. J. Mammal..

[40] Underwood W., Anthony R. (2020). AVMA guidelines for the euthanasia of animals: 2020 edition. Retrieved March.

[41] Sambrook J., Edward F.F., Tom M. (1989). Molecular Cloning: A Laboratory Manual.

[42] Agnarsson I., May-Collado L.J. (2008). The phylogeny of Cetartiodactyla: The importance of dense taxon sampling, missing data, and the remarkable promise of cytochrome b to provide reliable species-level phylogenies. Mol. Phylogenet. Evol..

[43] Tobe S.S., Kitchener A.C., Linacre A.M.T. (2010). Reconstructing Mammalian Phylogenies: A Detailed Comparison of the Cytochrome b and Cytochrome Oxidase Subunit I Mitochondrial Genes. PLoS ONE.

[44] Onditi K.O., Song W.Y., Li X.Y., Chen Z.Z., Li Q., He S.W., Musila S., Kioko E., Jiang X.L. (2022). Patterns and Predictors of Small Mammal Phylogenetic and Functional Diversity in Contrasting Elevational Gradients in Kenya. Front. Ecol. Evol..

[45] Sayers E.W., Cavanaugh M., Clark K., Ostell J., Pruitt K.D., Karsch-Mizrachi I. (2020). GenBank. Nucleic Acids Res..

[46] Edgar R.C. (2004). MUSCLE: Multiple sequence alignment with high accuracy and high throughput. Nucleic Acids Res..

[47] Talavera G., Castresana J. (2007). Improvement of phylogenies after removing divergent and ambiguously aligned blocks from protein sequence alignments. Syst. Biol..

[48] Steenwyk J.L., Buida T.J., Li Y., Shen X.-X., Rokas A. (2020). ClipKIT: A multiple sequence alignment trimming software for accurate phylogenomic inference. PLoS Biol..

[49] Vences M., Patmanidis S., Kharchev V., Renner S.S. (2022). Concatenator, a user-friendly program to concatenate DNA sequences, implementing graphical user interfaces for MAFFT and FastTree. Bioinform. Adv..

[50] Kalyaanamoorthy S., Minh B.Q., Wong T.K.F., von Haeseler A., Jermiin L.S. (2017). ModelFinder: Fast model selection for accurate phylogenetic estimates. Nat. Methods.

[51] Nguyen L.T., Schmidt H.A., von Haeseler A., Minh B.Q. (2015). IQ-TREE: A fast and effective stochastic algorithm for estimating maximum-likelihood phylogenies. Mol. Biol. Evol..

[52] Minh B.Q., Nguyen M.A., von Haeseler A. (2013). Ultrafast approximation for phylogenetic bootstrap. Mol. Biol. Evol..

[53] Bouckaert R., Vaughan T.G., Barido-Sottani J., Duchene S., Fourment M., Gavryushkina A., Heled J., Jones G., Kuhnert D., De Maio N. (2019). BEAST 2.5: An advanced software platform for Bayesian evolutionary analysis. PLoS Comput. Biol..

[54] Rambaut A., Drummond A.J. (2024). TreeAnnotator: MCMC Output Analysis, Version v2.7.7. https://www.beast2.org/treeannotator/.

[55] Ondřej M. (2018). Cutting tree branches to pick OTUs: A novel method of provisional species delimitation. bioRxiv.

[56] Kapli P., Lutteropp S., Zhang J., Kobert K., Pavlidis P., Stamatakis A., Flouri T. (2017). Multi-rate Poisson tree processes for single-locus species delimitation under maximum likelihood and Markov chain Monte Carlo. Bioinformatics.

[57] Fujisawa T., Barraclough T.G. (2013). Delimiting species using single-locus data and the Generalized Mixed Yule Coalescent approach: A revised method and evaluation on simulated data sets. Syst. Biol..

[58] Puillandre N., Brouillet S., Achaz G. (2021). ASAP: Assemble species by automatic partitioning. Mol. Ecol. Resour..

[59] Altschul S.F., Gish W., Miller W., Myers E.W., Lipman D.J. (1990). Basic local alignment search tool. J. Mol. Biol..

[60] Musser G.G., Carleton M.D., Wilson D.E., Reeder D.M. (2005). Superfamily Muroidea. Mammal Species of the World: A Taxonomic and Geographic Reference.

[61] Hutterer R., Wilson D.E., Reeder D.A.M. (2005). Order Soricomorpha. Mammal Species of the World: A Taxonomic and Geographic Reference.

[62] Wilson D.E., Mittermeier R.A., François T.L. (2019). Handbook of the Mammals of the World, Volume 8: Insectivores, Sloths and Colugos.

[63] Leigh J.W., Bryant D., Nakagawa S. (2015). popart: Full-feature software for haplotype network construction. Methods Ecol. Evol..

[64] Bandelt H.J., Forster P., Rohl A. (1999). Median-joining networks for inferring intraspecific phylogenies. Mol. Biol. Evol..

[65] Kimura M. (1980). A simple method for estimating evolutionary rates of base substitutions through comparative studies of nucleotide sequences. J. Mol. Evol..

[66] Hebert P.D., Cywinska A., Ball S.L., DeWaard J.R. (2003). Biological identifications through DNA barcodes. Proc. R. Soc. London. Ser. B Biol. Sci..

[67] Rozas J., Ferrer-Mata A., Sanchez-DelBarrio J.C., Guirao-Rico S., Librado P., Ramos-Onsins S.E., Sanchez-Gracia A. (2017). DnaSP 6: DNA Sequence Polymorphism Analysis of Large Data Sets. Mol. Biol. Evol..

[68] Tamura K., Stecher G., Kumar S. (2021). MEGA11: Molecular Evolutionary Genetics Analysis version 11. Mol. Biol. Evol..

[69] Suzuki H., Sato J.J., Tsuchiya K., Luo J., Zhang Y.-P., Wang Y.-X., Jiang X.-L. (2003). Molecular phylogeny of wood mice (Apodemus, Muridae) in East Asia. Biol. J. Linn. Soc..

[70] Rambaut A., Drummond A.J., Xie D., Baele G., Suchard M.A. (2018). Posterior Summarization in Bayesian Phylogenetics Using Tracer 1.7. Syst. Biol..

[71] Yu Y., Harris A.J., Blair C., He X. (2015). RASP (Reconstruct Ancestral State in Phylogenies): A tool for historical biogeography. Mol. Phylogenet. Evol..

[72] Matzke N.J. (2013). Probabilistic Historical Biogeography: New Models for Founder-Event Speciation, Imperfect Detection, and Fossils Allow Improved Accuracy and Model-Testing.

[73] Matzke N.J. (2014). Model selection in historical biogeography reveals that founder-event speciation is a crucial process in island clades. Syst. Biol..

[74] Ree R.H., Smith S.A. (2008). Maximum likelihood inference of geographic range evolution by dispersal, local extinction, and cladogenesis. Syst. Biol..

[75] Mousset S., Derome N., Veuille M. (2004). A Test of Neutrality and Constant Population Size Based on the Mismatch Distribution. Mol. Biol. Evol..

[76] Grant W.S. (2015). Problems and cautions with sequence mismatch analysis and Bayesian skyline plots to infer historical demography. J. Hered..

[77] Oksanen J., Blanchet F.G., Friendly M., Kindt R., Legendre P., McGlinn D., Minchin P.R., O’Hara R.B., Simpson G.L., Solymos P. (2019). vegan: Community Ecology Package, R Package Version 2.5-5. https://CRAN.R-project.org/package=vegan.

[78] Philipp B., Niklaus E.Z., Chantal H., Loïc P., Dirk Nikolaus K. (2022). CHELSA-BIOCLIM+ A Novel Set of Global Climate-Related Predictors at Kilometre-Resolution. EnviDat [Online].

[79] Hu W.-H., Onditi K.O., Jiang X.-L., Wu H.-L., Chen Z.-Z. (2022). Modeling the Potential Distribution of Two Species of Shrews (Chodsigoa hypsibia and Anourosorex squamipes) under Climate Change in China. Diversity.

[80] Dunne J.P., Horowitz L., Adcroft A., Ginoux P., Held I., John J., Krasting J.P., Malyshev S., Naik V., Paulot F. (2020). The GFDL Earth System Model version 4.1 (GFDL-ESM 4.1): Overall coupled model description and simulation characteristics. J. Adv. Model. Earth Syst..

[81] Riahi K., Rao S., Krey V., Cho C., Chirkov V., Fischer G., Kindermann G., Nakicenovic N., Rafaj P. (2011). RCP 8.5—A scenario of comparatively high greenhouse gas emissions. Clim. Chang..

[82] Schwalm C.R., Glendon S., Duffy P.B. (2020). RCP 8.5 tracks cumulative CO2 emissions. Proc. Natl. Acad. Sci. USA.

[83] Hijmans R.J. (2024). terra: Spatial Data Analysis, R Package Version 1.7-83. https://CRAN.R-project.org/package=terra.

[84] Kass J.M., Vilela B., Aiello-Lammens M.E., Muscarella R., Merow C., Anderson R.P. (2018). Wallace: A flexible platform for reproducible modeling of species niches and distributions built for community expansion. Methods Ecol. Evol..

[85] Kass J.M., Pinilla-Buitrago G.E., Paz A., Johnson B.A., Grisales-Betancur V., Meenan S.I., Attali D., Broennimann O., Galante P.J., Maitner B.S. (2023). wallace 2: A shiny app for modeling species niches and distributions redesigned to facilitate expansion via module contributions. Ecography.

[86] Phillips S.J., Anderson R.P., Schapire R.E. (2006). Maximum entropy modeling of species geographic distributions. Ecol. Model..

[87] Li X., Yu L., Sohl T.L., Clinton N., Li W., Zhu Z., Liu X., Gong P. (2016). A cellular automata downscaling based 1 km global land use datasets (2010–2100). Sci. Bull..

[88] Demos T.C., Agwanda B., Hickerson M.J. (2013). Integrative taxonomy within the Hylomyscus denniae complex (Rodentia: Muridae) and a new species from Kenya. J. Mammal..

[89] Morgan K., Mboumba J.-F., Ntie S., Mickala P., Miller C., Zhen Y., Harrigan R., Underwood V., Ruegg K., Fokam E. (2020). Precipitation and vegetation shape patterns of genomic and craniometric variation in the central African rodent Praomys misonnei. Proc. R. Soc. B Biol. Sci..

[90] Mairal M., Sanmartin I., Herrero A., Pokorny L., Vargas P., Aldasoro J.J., Alarcon M. (2017). Geographic barriers and Pleistocene climate change shaped patterns of genetic variation in the Eastern Afromontane biodiversity hotspot. Sci. Rep..

[91] Genung M.A., Schweitzer J.A., Úbeda F., Fitzpatrick B.M., Pregitzer C.C., Felker-Quinn E., Bailey J.K. (2011). Genetic variation and community change—Selection, evolution, and feedbacks. Funct. Ecol..

[92] Allendorf F.W. (1986). Genetic drift and the loss of alleles versus heterozygosity. Zoo. Biol..

[93] Barton N.H., Charlesworth B. (1984). Genetic revolutions, founder effects, and speciation. Annu. Rev. Ecol. Syst..

[94] Romiguier J., Gayral P., Ballenghien M., Bernard A., Cahais V., Chenuil A., Chiari Y., Dernat R., Duret L., Faivre N. (2014). Comparative population genomics in animals uncovers the determinants of genetic diversity. Nature.

[95] Naciri Y., Linder H.P. (2020). The genetics of evolutionary radiations. Biol. Rev..

[96] Bragg J.G., Supple M.A., Andrew R.L., Borevitz J.O. (2015). Genomic variation across landscapes: Insights and applications. New Phytol..

[97] Hand B.K., Lowe W.H., Kovach R.P., Muhlfeld C.C., Luikart G. (2015). Landscape community genomics: Understanding eco-evolutionary processes in complex environments. Trends Ecol. Evol..

[98] Mallet J., Besansky N., Hahn M.W. (2016). How reticulated are species?. Bioessays.

[99] Slatkin M. (1987). Gene flow and the geographic structure of natural populations. Science.

[100] Cai L., Xi Z., Lemmon E.M., Lemmon A.R., Mast A., Buddenhagen C.E., Liu L., Davis C.C. (2020). The Perfect Storm: Gene Tree Estimation Error, Incomplete Lineage Sorting, and Ancient Gene Flow Explain the Most Recalcitrant Ancient Angiosperm Clade, Malpighiales. Syst. Biol..

[101] Bayne B.L. (2017). Chapter 1—Phylogeny. Developments in Aquaculture and Fisheries Science.

[102] Schoville S.D., Roderick G.K., Kavanaugh D.H. (2012). Testing the ‘Pleistocene species pump’ in alpine habitats: Lineage diversification of flightless ground beetles (Coleoptera: Carabidae: Nebria) in relation to altitudinal zonation. Biol. J. Linn. Soc..

[103] Janzen T., Etienne R.S. (2017). Inferring the role of habitat dynamics in driving diversification: Evidence for a species pump in Lake Tanganyika cichlids. bioRxiv.

[104] Kimura M., Weiss G.H. (1964). The Stepping Stone Model of Population Structure and the Decrease of Genetic Correlation with Distance. Genetics.

[105] Slatkin M. (1993). Isolation by Distance in Equilibrium and Non-Equilibrium Populations. Evolution.

[106] Morley C., Harper R., Wigger S., Morley C. (1999). Introduction to the East African Rift System. Geoscience of Rift Systems—Evolution of East Africa: AAPG Studies in Geology.

[107] Nicolas V., Wendelen W., Barriere P., Dudu A., Colyn M. (2008). Morphometric variation in Hylomyscus alleni and H. stella (Rodentia: Muridae), and description of a new species. J. Mammal..

[108] Nicolas V., Querouil S., Verheyen E., Verheyen W., Mboumba J.F., Dillen M., Colyn M. (2006). Mitochondrial phylogeny of African wood mice, genus Hylomyscus (Rodentia, Muridae): Implications for their taxonomy and biogeography. Mol. Phylogenet. Evol..

[109] Carleton M.D., Kerbis Peterhans J.C., Kerbis Peterhans J.C., Stanley W.T., Graves G.R. (2006). Review of the Hylomyscus denniae group (Rodentia: Muridae) in eastern Africa, with comments on the generic allocation of Epimys endorobae Heller. Proc. Biol. Soc. Wash..

[110] Carleton M.D., Stanley W.T., Graves G.R. (2005). Review of the Hylomyscus denniae complex (Rodentia: Muridae) in Tanzania, with a description of a new species. Proc. Biol. Soc. Wash..

[111] Lapin K., Hoffmann J.A., Braun M., Oettel J. (2024). Identification and prioritization of stepping stones for biodiversity conservation in forest ecosystems. Conserv. Sci. Pract..

[112] Theodoridis S., Fordham D.A., Brown S.C., Li S., Rahbek C., Nogues-Bravo D. (2020). Evolutionary history and past climate change shape the distribution of genetic diversity in terrestrial mammals. Nat. Commun..

[113] Bennun L.A., Njoroge P. (1999). Important Bird Areas in Kenya.

[114] Bradshaw C.J.A., Sodhi N.S., Brook B.W. (2008). Tropical turmoil: A biodiversity tragedy in progress. Front. Ecol. Environ..

[115] Chrisphine O., Odhiambo A., Boitt K. (2016). Assessment of hydrological impacts of Mau Forest, Kenya. Hydrol. Curr. Res..

[116] Gichuhi M. (2013). Ecological management of the Mau catchment area and its impact on Lake Nakuru national park. J. Agric. Sci. Technol..

[117] Karanja F., Tessema Y., Barrow E.G. (2002). Equity in the Loita/Purko Naimina Enkiyio Forest in Kenya: Securing Maasai Rights to and Responsibilities for the Forest.

[118] Kundu P.M., Olang L.O. (2011). Automated extraction of morphologic and hydrologic properties for River Njoro catchment in Eastern Mau, Kenya. AGSE.

[119] Maundu P., Berger D., Ole Saitabau C., Nasieku J., Kipelian M., Mathenge S., Morimoto Y., Höft R. (2001). Ethnobotany of the Loita Maasai. People and Plants Working Paper.

[120] Mbuvi M.T., Musyoki J.K., Ongugo P.O. (2015). Equity Mechanisms in traditional forest management Systems: A case study of Loita forest in Kenya. J. Sustain. For..

[121] Olang L.O., Kundu P.M. (2011). Land degradation of the Mau forest complex in Eastern Africa: A review for management and restoration planning. Environ. Monit..

[122] Tsingalia M.H. (1988). Animals and the Regeneration of an African Rainforest Tree.

[123] Allaire J.J., Xie Y., Dervieux C., McPherson J., Luraschi J., Ushey K., Atkins A. (2024). Rmarkdown: Dynamic Documents for r (Version R Package 2.28). https://github.com/rstudio/rmarkdown.

[124] Boettiger C. (2021). Knitcitations: Citations for ‘Knitr’ Markdown Files (Version R Package 1.0.12). https://CRAN.R-project.org/package=knitcitations.

[125] Hijmans R.J. (2023). Raster: Geographic Data Analysis and Modeling (Version R Package 3.6-26). https://CRAN.R-project.org/package=raster.

[126] Merow C., Maitner B., Owens H., Kass J., Enquist B., Guralnik R., Zurrell D., Koenig C. (2023). rangeModelMetadata: Provides Templates for Metadata Files Associated with Species Range Models (Version R Package 0.1.5). https://CRAN.R-project.org/package=rangeModelMetadata.

[127] Xie Y., Stodden V., Leisch F., Peng R.D. (2014). Knitr: A Comprehensive Tool for Reproducible Research in R. Implementing Reproducible Computational Research.

[128] (2015). Dynamic Documents with R and Knitr.

[129] (2024). Knitr: A General-Purpose Package for Dynamic Report Generation in r (Version R Package 1.48). https://yihui.org/knitr/.

[130] Xie Y., Allaire J.J., Grolemund G. (2018). R Markdown: The Definitive Guide.

[131] Xie Y. (2020). Christophe Dervieux, and Emily Riederer. R Markdown Cookbook.

[132] He K., Li Y.J., Brandley M.C., Lin L.K., Wang Y.X., Zhang Y.P., Jiang X.L. (2010). A multi-locus phylogeny of Nectogalini shrews and influences of the paleoclimate on speciation and evolution. Mol. Phylogenet. Evol..

[133] Stanhope M.J., Smith M.R., Waddell V.G., Porter C.A., Shivji M.S., Goodman M. (1996). Mammalian evolution and the interphotoreceptor retinoid binding protein (IRBP) gene: Convincing evidence for several superordinal clades. J. Mol. Evol..

