# Supplementary material for: Comparative Phylogeography of Two Specialist Rodents in Forest Fragments in Kenya
Source: Life (Basel). 2024 Nov 12;14(11):1469. doi: 10.3390/life14111469 (PMC11595787; doi:10.3390/life14111469)
Supplement: Supplementary file 1 [file life-14-01469-s001.zip › Supplementary File S1.pdf]

Supplementary

Comparative Phylogeography of Two Specialist Rodents in Forest Fragments in Kenya

Alois Wambua Mweu [1,2,†], Kenneth Otieno Onditi [1,2,3,*,†], Laxman Khanal [4], Simon Musila [2], Esther Kioko [2] and Xuelong Jiang [1,3,*]

1 Key Laboratory of Genetic Evolution and Animal Models, Kunming Institute of Zoology, Chinese Academy of Sciences, Kunming 650201, China; aliwambua@gmail.com
2 Zoology Section, National Museums of Kenya, Nairobi P.O. Box 40658-00100, Kenya
3 Sino-Africa Joint Research Centre, Chinese Academy of Sciences, Nairobi P.O. Box 62000-00200, Kenya
4 Central Department of Zoology, Institute of Science and Technology, Tribhuvan University, Kathmandu 44618, Nepal; lkhanal@cdztu.edu.np
* Correspondence: kenneth@mail.kiz.ac.cn (K.O.O.); jiangxl@mail.kiz.ac.cn (X.J.)
† These authors contributed equally to this work.

Supplementary File S1: Extended study areas' descriptions

Material and Methods

Study area

Samples were sourced from fieldwork conducted in Kenya by joint zoological survey teams from the Kunming Institute of Zoology (KIZ) and the National Museums of Kenya (NMK) between 2015 and 2018 (**Error! Reference source not found.**). These surveys yielding several *Hylomyscus endorobae* and *Praomys jacksoni* samples. Mount Kenya was surveyed between 6th September 2015 and 13th October 2015, Loita Hills between 9th January and 5th February 2018, Mau Forest from 12th August to 3rd September 2018, and Kakamega Forest from 5th to 14th September 2018. Additionally, we scoured the available literature and obtained additional data on these two species from the Western Kenya Mt. Elgon and Cherangani Hills, the Aberdares

Ranges, and outlier Mau Forest Fragments, thus bridging geographical coverages in sampling the critical montane highland ecosystems across the known range of these two lineages in Kenya. Key orographic and geomorphological characteristics define these ecosystems (Mahaney, 1988; Mahaney *et al.*, 1991; Case, 2006; Michael, 2006; Jaramillo *et al.*, 2011; Schüler *et al.*, 2012; Leclerc *et al.*, 2014; Maslin *et al.*, 2014; Menegon *et al.*, 2014; Peyron *et al.*, 2017; Liu *et al.*, 2018).

Mount Kenya (0°08'59"S 37°18'28"E) is Africa's second-highest peak, emerged approximately three million years ago from the volcanic activities of the East African Rift, which occurred just before the last Ice Age. This extinct volcano is believed to have erupted about 3 million years ago during the Pliocene epoch. Its formation is associated with the East African Rift System, a significant tectonic structure that influenced volcanic activity. The uplift linked to the rift system brought about substantial changes in the regional climate and hydrology, impacting the ecological zones in the surrounding areas. As a biodiversity hotspot, Mt. Kenya supports unique alpine and sub-alpine ecosystems. It is a crucial area for studying high-altitude adaptation in plants and animals and plays a role in the distribution and evolution of vertebrate species through both vicariance and dispersal. It plays a crucial role as a water catchment area, significantly benefiting local communities and agricultural endeavors throughout Kenya, besides being critical to wildlife conservation efforts (Bennun & Njoroge, 1999). Mount Kenya's varied elevations feature distinct yet interconnected habitats that evolve from lush evergreen forests and bamboo zones to scrublands and moorlands. As one climbs higher, these give way to rocky terrains and reduced patches of ice and snow, a diversity influenced by factors like altitude, rainfall patterns, soil composition, slope orientation, and human activities. The lower slopes are adorned with a rich tapestry of indigenous forests, which, based on their position relative to

prevailing winds, transition into bamboo zones and then into montane grasslands and heather or alpine zones.

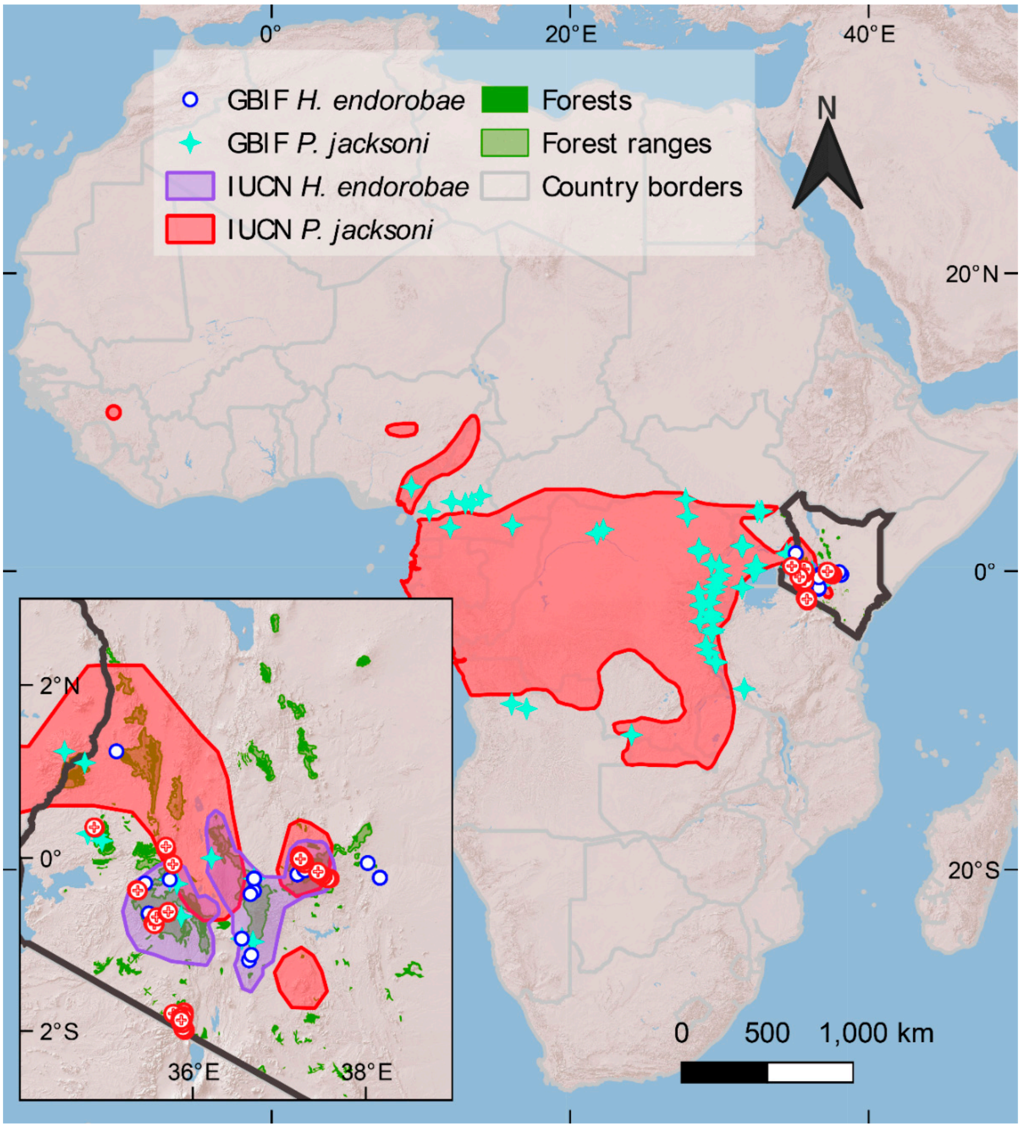

Distribution maps of *Praomys jacksoni* (red shaded regions [International Union for Conservation of Nature (IUCN) Red List records] and cyan stars [Global Biodiversity Information Facility (GBIF) records] and *Hylomyscus endorobae* (purple shaded regions [IUCN records] and [white blue-outlined circles [GBIF records] and the new sampling sites in Kenya

[red-outlined circles with a red plus sign]. The inset map zooms in on the Kenya distribution and the new survey sites.

The Mau Forest Complex (0°39'31"S 35°31'14"E) is the largest indigenous forest, extending over 400 km² with altitudes ranging from 1,200 to 3,000 meters located on the Mau Escarpment within the Great Rift Valley system, constitutes the largest remaining indigenous montane forest in East Africa. Their rich history dates back to the late Pleistocene and Holocene epochs. These forests are essential in regulating the regional climate and are a significant catchment area, impacting the water supply to various lakes and rivers in the Rift Valley. It is the nation's largest water tower and closed-canopy forest ecosystem in Eastern Africa and an important water catchment in Kenya (Kundu & Olang, 2011; Olang & Kundu, 2011). The Mau Forest is integral for water regulation and serves as a sanctuary for diverse wildlife, playing a critical role in maintaining local and regional ecological balance and supporting human livelihoods. The Mau Forest complex is the largest of Kenya's watersheds (i.e., among Mau Forest Complex, Mt. Kenya, Aberdares, Cherangany Hills, and Mt. Elgon) and the largest closed-canopy montane ecosystem in Eastern Africa (Chrisphine *et al.*, 2016). In the past three decades the forest complex has undergone significant land use changes due to increased human population demanding land for settlement and subsistence agriculture, that has led to considerable land fragmentation and deforestation of the headwater catchments (Olang & Kundu, 2011).

The Loita Hills (1°45'34"S 35°58'39"E; ~1,000-2,300 meters; 300 km²) primarily consist of a chain of isolated hills and a range of hills in Loita-Narok Kenya. They are an important forest in Kenya that has received little attention in biodiversity surveys despite being a large forest ecosystem (approximately 330 km^2), and not legally protected (Mbuvi *et al.*, 2015). These

hills are crucial in moderating the local climate and serving as a water catchment area, and conserving forest ecosystems and species, including unique highland ecosystem, various plant and animal species, and several rare bird species. The forest is communally owned by members of the Loita Maasai community, who use it mainly for animal grazing and extraction of medicinal plants (Maundu *et al.*, 2001; Karanja *et al.*, 2002). Because of the large numbers of livestock animals by many community members, competition for grazing areas is high in these forests, which affects forest regeneration and, hence, the health of the entire forest. In addition, a sizeable proportion of local people around these forests is gradually turning to crop cultivation, which has resulted in clearing some forests to open them for farming (Maundu *et al.*, 2001). Although the majority of the Loita Hills Forests are highly threatened, little research has been undertaken on them except that of plants [14], and any attempt by the government to gazette some of the forests in Loita to reserves to safeguard their future has been rejected by local Masai community (Maundu *et al.*, 2001). With continuous human population increase and the demand for land for cultivation, cattle overgrazing, human settlements, and infrastructure development, deforestation will likely continue, causing an extinction crisis among tropical forest species (Bradshaw *et al.*, 2008).

The Kakamega Forest (0°16'33"N 34°53'20"E; 1,466 to 1,779 meters) is Kenya's only tropical rainforest, covering an area of roughly 238 square kilometers, of which less than half is pristine indigenous forest. This forest serves as a relic of the Guineo-Congolian rainforest with an extensive history exceeding 2 million years. Unique among East African forests, Kakamega offers a critical ecological and evolutionary niche of significant importance. It plays a fundamental role in shaping the local climate and water cycle, thereby bolstering the biodiversity of the region. The forest hosts an array of exclusive flora and fauna, establishing itself as a

pivotal site for delving into tropical rainforest ecology and the evolutionary processes of forest species. Kakamega Forest positions itself as the final eastern boundary of the ancient Guinea-Congolian rainforest that once covered the continent, standing today as an indispensable ecological marvel venerated for its abundant biodiversity and its status as a symbol of the country's rainforest legacy (Tsingalia, 1988; Gichuhi, 2013).

Mount Elgon, an extensive extinct shield volcano, is distinguished by its long geological history, with initial eruptions traced back approximately 24 million years ago. This venerable geological feature is among the earliest volcanic formations in the East African landscape. Its historical volcanic activities are intricately connected to the complex tectonic movements of the East African Rift. The sprawling caldera of Mount Elgon, combined with its fertile volcanic soils, significantly enriches the area's biodiversity. It also plays a pivotal role in influencing the local climate conditions. Encompassing an array of habitats that range from verdant lowland forests to rugged high moorlands, Mount Elgon offers invaluable insights into montane ecology. It serves as a living laboratory for studying evolutionary processes that shape mountain biomes across the globe. On the other hand, the Cherangani Hills, a series of highlands dissected by deep valleys in the northwestern segment of the Rift Valley, hold geological significance. These hills, which date back to the Miocene epoch, originate from volcanic activities and are intimately related to the uplifting forces of the Rift Valley's formation. Their presence significantly influences local weather patterns and plays a crucial role in the hydrology of the region, acting as sources for several major rivers. The Cherangani Hills are renowned for their vast diversity of forest types, each supporting unique ecological communities.

List of References

Bennun, L. A., & Njoroge, P. (1999). *Important bird areas in Kenya*.

Bradshaw, C. J. A., Sodhi, N. S., & Brook, B. W. (2008). Tropical turmoil: a biodiversity tragedy in progress. *Frontiers in Ecology and the Environment*, *7*(2), 79-87. doi: 10.1890/070193

Case, M. (2006). *Climate change impacts on East Africa*. W.-W. W. F. F. N. f. W. W. Fund).

Chrisphine, O., Odhiambo, A., & Boitt, K. (2016). Assessment of hydrological impacts of Mau Forest, Kenya. *Hydrology: Current Research*, *7*(1). doi:

Gichuhi, M. (2013). Ecological management of the Mau catchment area and its impact on Lake Nakuru national park. *Journal of Agriculture, Science and Technology*, *15*(1), 81-101. doi:

Jaramillo, J., Muchugu, E., Vega, F. E., Davis, A., Borgemeister, C., & Chabi-Olaye, A. (2011). Some like it hot: the influence and implications of climate change on coffee berry borer (Hypothenemus hampei) and coffee production in East Africa. *PLoS ONE*, *6*(9), e24528. doi: 10.1371/journal.pone.0024528

Karanja, F., Tessema, Y., & Barrow, E. G. (2002). *Equity in the Loita/Purko Naimina Enkiyio forest in Kenya: Securing Maasai rights to and responsibilities for the forest*. IUCN.

Kundu, P. M., & Olang, L. O. (2011). Automated extraction of morphologic and hydrologic properties for River Njoro catchment in Eastern Mau, Kenya. *AGSE 2011*, 147. doi:

Leclerc, C., Mwongera, C., Camberlin, P., & Moron, V. (2014). Cropping System Dynamics, Climate Variability, and Seed Losses among East African Smallholder Farmers: A

Retrospective Survey. *Weather, Climate, and Society*, *6*(3), 354-370. doi: 10.1175/wcas-d-13-00035.1

Liu, X., Rendle-Bühring, R., & Henrich, R. (2018). High-and low-latitude forcing of the East African climate since the LGM: Inferred from the elemental composition of marine sediments off Tanzania. *Quaternary Science Reviews*, *196*, 124-136. doi: 10.1016/j.quascirev.2018.08.004

Mahaney, W. (1988). Holocene glaciations and paleoclimate of mount Kenya and other East African mountains. *Quaternary Science Reviews*, *7*(2), 211-225. doi: 10.1016/0277-3791(88)90007-8

Mahaney, W. C., Harmsen, R., & Spence, J. R. (1991). Glacial-interglacial cycles and development of the Afroalpine ecosystem on East African Mountains: I. Glacial and postglacial geological record and paleoclimate of Mount Kenya. *Journal of African Earth Sciences (and the Middle East)*, *12*(3), 505-512. doi: 10.1016/0899-5362(91)90142-l

Maslin, M. A., Brierley, C. M., Milner, A. M., Shultz, S., Trauth, M. H., & Wilson, K. E. (2014). East African climate pulses and early human evolution. *Quaternary Science Reviews*, *101*(-), 1-17. doi: 10.1016/j.quascirev.2014.06.012

Maundu, P., Berger, D., Ole Saitabau, C., Nasieku, J., Kipelian, M., Mathenge, S., Morimoto, Y., & Höft, R. (2001). Ethnobotany of the Loita Maasai. *People and plants working paper*, *8*. doi:

Mbuvi, M. T., Musyoki, J. K., & Ongugo, P. O. (2015). Equity Mechanisms in traditional forest management Systems: a case study of Loita forest in Kenya. *Journal of Sustainable Forestry*, *34*(4), 380-405. doi:

Menegon, M., Loader, S. P., Marsden, S. J., Branch, W. R., Davenport, T. R., & Ursenbacher, S. (2014). The genus Atheris (Serpentes: Viperidae) in East Africa: phylogeny and the role of rifting and climate in shaping the current pattern of species diversity. *Mol Phylogenet Evol*, *79*, 12-22. doi: 10.1016/j.ympev.2014.06.007

Michael, C. (2006). Climate change impacts on East Africa a review of the scientific literature. *WFF-World Wide Fund For Nature, Gland, Switzerland*. doi:

Olang, L. O., & Kundu, P. M. (2011). Land degradation of the Mau forest complex in Eastern Africa: a review for management and restoration planning. *Environmental Monitoring*, *15*, 245-262. doi:

Peyron, O., Jolly, D., Bonnefille, R., Vincens, A., & Guiot, J. (2017). Climate of East Africa 6000 14C Yr B.P. as Inferred from Pollen Data. *Quaternary Research*, *54*(1), 90-101. doi: 10.1006/qres.2000.2136

Schüler, L., Hemp, A., Zech, W., & Behling, H. (2012). Vegetation, climate and fire-dynamics in East Africa inferred from the Maundi crater pollen record from Mt Kilimanjaro during the last glacial–interglacial cycle. *Quaternary Science Reviews*, *39*, 1-13. doi: 10.1016/j.quascirev.2012.02.003

Tsingalia, M. H. (1988). *Animals and the regeneration of an African rainforest tree*. University of California, Berkeley.
