# Supplementary material for: Comparative Phylogeography of Two Specialist Rodents in Forest Fragments in Kenya
Source: Life (Basel). 2024 Nov 12;14(11):1469. doi: 10.3390/life14111469 (PMC11595787; doi:10.3390/life14111469)
Supplement: Supplementary file 1 [file life-14-01469-s001.zip › Supplementary File S2.pdf]

Supplementary

Comparative Phylogeography of Two Specialist Rodents in Forest Fragments in Kenya

Alois Wambua Mweu [1,2,†], Kenneth Otieno Onditi [1,2,3,*,†], Laxman Khanal [4], Simon Musila [2], Esther Kioko [2] and Xuelong Jiang [1,3,*]

[1] Key Laboratory of Genetic Evolution and Animal Models, Kunming Institute of Zoology, Chinese Academy of Sciences, Kunming 650201, China; aliwambua@gmail.com
[2] Zoology Section, National Museums of Kenya, Nairobi P.O. Box 40658-00100, Kenya
[3] Sino-Africa Joint Research Centre, Chinese Academy of Sciences, Nairobi P.O. Box 62000-00200, Kenya
[4] Central Department of Zoology, Institute of Science and Technology, Tribhuvan University, Kathmandu 44618, Nepal; lkhanal@cdztu.edu.np
[*] Correspondence: kenneth@mail.kiz.ac.cn (K.O.O.); jiangxl@mail.kiz.ac.cn (X.J.)
[†] These authors contributed equally to this work.

Supplementary File S2: Species distribution modeling data processing

This document was reproduced from the data preparation stages in Wallace (Kass, 2018;Kass, 2023) prior to the species distribution modelling in Maxent.

Package installation

```
library(spocc)
library(spThin)
library(dismo)
library(sf)
library(ENMeval)
library(wallace)
```

Analysis for *Hylomyscus endorobae* (He)

Obtain Occurrence Data

You searched the gbif database for *Hylomyscus endorobae*, limited to 9.99999^{5} records. You decided to remove occurrences without uncertainty information? FALSE

```
# Query selected database for occurrence records
queryDb_He <- occs_queryDb(
  spNames = "Hylomyscus endorobae",
  occDb = "gbif",
  occNum = 999999,
  RmUncertain = FALSE)
occs_He <- queryDb_He$Hylomyscus_endorobae$cleaned
```

Obtain environmental data

Using user-specified variables.

```
## Specify the directory with the environmental variables
dir_envs_He <- ""
envs_path <- file.path(dir_envs_He, c('bio01.tif', 'bio02.tif', 'bio03.tif',
'bio04.tif', 'bio05.tif', 'bio06.tif', 'bio07.tif', 'bio08.tif', 'bio09.tif',
'bio10.tif', 'bio11.tif', 'bio12.tif', 'bio13.tif', 'bio14.tif', 'bio15.tif',
'bio16.tif', 'bio17.tif', 'bio18.tif', 'bio19.tif'))
# Create environmental object
envs_He <- envs_userEnvs(
  rasPath = envs_path,
  rasName = c('bio01.tif', 'bio02.tif', 'bio03.tif', 'bio04.tif', 'bio05.tif',
'bio06.tif', 'bio07.tif', 'bio08.tif', 'bio09.tif', 'bio10.tif', 'bio11.tif',
'bio12.tif', 'bio13.tif', 'bio14.tif', 'bio15.tif', 'bio16.tif', 'bio17.tif',
'bio18.tif', 'bio19.tif'),
  doBrick = TRUE)
occs_xy_He <- occs_He[c('longitude', 'latitude')]
occs_vals_He <- as.data.frame(raster::extract(envs_He, occs_xy_He, cellnumbers =
TRUE))
# Remove duplicated same cell values
occs_He <- occs_He[!duplicated(occs_vals_He[, 1]), ]
occs_vals_He <- occs_vals_He[!duplicated(occs_vals_He[, 1]), -1]
# remove occurrence records with NA environmental values
occs_He <- occs_He[!(rowSums(is.na(occs_vals_He)) >= 1), ]
# also remove variable value rows with NA environmental values
occs_vals_He <- na.omit(occs_vals_He)
# add columns for env variable values for each occurrence record
occs_He <- cbind(occs_He, occs_vals_He)
```

Process Occurrence Data

Remove the occurrence localities by ID.

```
# remove the rows that match the occIDs selected
occs_He <- poccs_removeByID(
  occs = occs_He ,
  removeID = 163)
```

Process Occurrence Data

Thinning the occurrences to 10 km

```
# Thin occurrences
occs_He <- poccs_thinOccs(
  occs = occs_He,
  thinDist = 10)
```

Process environmental data

Sampling of 10000 background points and corresponding environmental data using a “minimum convex polygon” method with a 2 degree buffer.

```
# Generate background extent
bgExt_He <- penvs_bgExtent(
```

```
  occs = occs_He,
  bgSel = "minimum convex polygon",
  bgBuf = 2)
# Mask environmental data to provided extent
bgMask_He <- penvs_bgMask(
  occs = occs_He,
  envs = envs_He,
  bgExt = bgExt_He)
# Sample background points from the provided area
bgSample_He <- penvs_bgSample(
  occs = occs_He,
  bgMask =  bgMask_He,
  bgPtsNum = 10000)
# Extract values of environmental layers for each background point
bgEnvsVals_He <- as.data.frame(raster::extract(bgMask_He,  bgSample_He))
##Add extracted values to background points table
bgEnvsVals_He <- cbind(scientific_name = paste0("bg_", "Hylomyscus endorobae"),
bgSample_He,
                            occID = NA, year = NA, institution_code = NA, country =
NA,
                            state_province = NA, locality = NA, elevation = NA,
                            record_type = NA, bgEnvsVals_He)
```

Partition occurrence data

Partition occurrences and background points for model training and validation using “spatial block”, a spatial partition method with an aggregation factor of 2.

```
# R code to get partitioned data
groups_He <- part_partitionOccs(
  occs = occs_He ,
  bg =  bgSample_He,
  method = "block",
  bgMask = bgMask_He,
  aggFact = 2)
```

Build and Evaluate Niche Model

Generating a species distribution model using the maxnet algorithm as implemented in ENMeval V2.0 (with clamping = TRUE). For tuning using L feature classes and regularization multipliers in the 1, 2 range increasing by 1. Not using any categorical predictor variables.

```
# Run maxent model for the selected species
model_He <- model_maxent(
  occs = occs_He,
  bg = bgEnvsVals_He,
  user.grp = groups_He,
  bgMsk = bgMask_He,
  rms = c(1, 2),
  rmsStep =  1,
  fcs = 'L',
  clampSel = TRUE,
  algMaxent = "maxnet",
  parallel = FALSE,
  numCores = 15)
```

Analysis for *Praomys jacksoni* (Pj)

Obtain Occurrence Data

You searched the gbif database for *Praomys jacksoni*, limited to 9.99999^{5} records. You decided to remove occurrences without uncertainty information? FALSE

```
# Query selected database for occurrence records
queryDb_Pj <- occs_queryDb(
  spNames = "Praomys jacksoni",
  occDb = "gbif",
  occNum = 999999,
  RmUncertain = FALSE)
occs_Pj <- queryDb_Pj$Praomys_jacksoni$cleaned
```

Obtain environmental data

Using user-specified variables.

```
## Specify the directory with the environmental variables
dir_envs_Pj <- ""
envs_path <- file.path(dir_envs_Pj, c('bio01.tif', 'bio02.tif', 'bio03.tif',
'bio04.tif', 'bio05.tif', 'bio06.tif', 'bio07.tif', 'bio08.tif', 'bio09.tif',
'bio10.tif', 'bio11.tif', 'bio12.tif', 'bio13.tif', 'bio14.tif', 'bio15.tif',
'bio16.tif', 'bio17.tif', 'bio18.tif', 'bio19.tif'))
# Create environmental object
envs_Pj <- envs_userEnvs(
  rasPath = envs_path,
  rasName = c('bio01.tif', 'bio02.tif', 'bio03.tif', 'bio04.tif', 'bio05.tif',
'bio06.tif', 'bio07.tif', 'bio08.tif', 'bio09.tif', 'bio10.tif', 'bio11.tif',
'bio12.tif', 'bio13.tif', 'bio14.tif', 'bio15.tif', 'bio16.tif', 'bio17.tif',
'bio18.tif', 'bio19.tif'),
  doBrick = TRUE)
occs_xy_Pj <- occs_Pj[c('longitude', 'latitude')]
occs_vals_Pj <- as.data.frame(raster::extract(envs_Pj, occs_xy_Pj, cellnumbers =
TRUE))
# Remove duplicated same cell values
occs_Pj <- occs_Pj[!duplicated(occs_vals_Pj[, 1]), ]
occs_vals_Pj <- occs_vals_Pj[!duplicated(occs_vals_Pj[, 1]), -1]
# remove occurrence records with NA environmental values
occs_Pj <- occs_Pj[!(rowSums(is.na(occs_vals_Pj)) >= 1), ]
# also remove variable value rows with NA environmental values
occs_vals_Pj <- na.omit(occs_vals_Pj)
# add columns for env variable values for each occurrence record
occs_Pj <- cbind(occs_Pj, occs_vals_Pj)
```

Process Occurrence Data

Thinning the occurrences to 10 km

```
# Thin occurrences
occs_Pj <- poccs_thinOccs(
  occs = occs_Pj,
  thinDist = 10)
```

Process environmental data

Sampling of 10000 background points and corresponding environmental data using a "minimum convex polygon" method with a 2-degree buffer.

```
# Generate background extent
bgExt_Pj <- penvs_bgExtent(
  occs = occs_Pj,
  bgSel = "minimum convex polygon",
  bgBuf = 2)


# Mask environmental data to provided extent
bgMask_Pj <- penvs_bgMask(
  occs = occs_Pj,
  envs = envs_Pj,
  bgExt = bgExt_Pj)


# Sample background points from the provided area
bgSample_Pj <- penvs_bgSample(
  occs = occs_Pj,
  bgMask =  bgMask_Pj,
  bgPtsNum = 10000)


# Extract values of environmental layers for each background point
bgEnvsVals_Pj <- as.data.frame(raster::extract(bgMask_Pj,  bgSample_Pj))


##Add extracted values to background points table
bgEnvsVals_Pj <- cbind(scientific_name = paste0("bg_", "Praomys jacksoni"),
bgSample_Pj, occID = NA, year = NA, institution_code = NA, country = NA,
state_province = NA, locality = NA, elevation = NA, record_type = NA, bgEnvsVals_Pj)
```

Partition occurrence data

Partition occurrences and background points for model training and validation using "spatial block", a spatial partition method with an aggregation factor of 2.

```
# R code to get partitioned data
groups_Pj <- part_partitionOccs(
  occs = occs_Pj ,
  bg =  bgSample_Pj,
  method = "block",
  bgMask = bgMask_Pj,
  aggFact = 2)
```

Build and Evaluate Niche Model

Generating a species distribution model using the maxnet algorithm as implemented in ENMeval V2.0 (with clamping = TRUE). For tuning using L feature classes and regularization multipliers in the 1, 2 range increasing by 1. Not using any categorical predictor variables.

```
# Run maxent model for the selected species
model_Pj <- model_maxent(
```

```
  occs = occs_Pj,
  bg = bgEnvsVals_Pj,
  user.grp = groups_Pj,
  bgMsk = bgMask_Pj,
  rms = c(1, 2),
  rmsStep =  1,
  fcs = 'L',
  clampSel = TRUE,
  algMaxent = "maxnet",
  parallel = FALSE,
  numCores = 15)
```

References

Allaire, JJ, Yihui Xie, Christophe Dervieux, Jonathan McPherson, Javier Luraschi, Kevin Ushey, Aron Atkins, et al. 2024. *Rmarkdown: Dynamic Documents for r* (version R package 2.28). https://github.com/rstudio/rmarkdown.

Boettiger, Carl. 2021. *Knitcitations: Citations for 'Knitr' Markdown Files* (version R package 1.0.12). https://CRAN.R-project.org/package=knitcitations.

Hijmans, Robert J. 2023. *Raster: Geographic Data Analysis and Modeling* (version R package 3.6-26). https://CRAN.R-project.org/package=raster.

J. M. Kass, G. E. Pinilla-Buitrago, A. Paz, B. A. Johnson, V. Grisales-Betancur, S. I. Meenan, D. Attali, et al. 2023. "Wallace 2: A Shiny App for Modeling Species Niches and Distributions Redesigned to Facilitate Expansion via Module Contributions." *Ecography* 2023(3) (e06547): 1–9. https://onlinelibrary.wiley.com/doi/10.1111/ecog.06547.

Merow, Cory, Brian Maitner, Hannah Owens, Jamie Kass, Brian Enquist, Rob Guralnik, Damaris Zurrell, and Christian Koenig. 2023. *rangeModelMetadata: Provides Templates for Metadata Files Associated with Species Range Models* (version R package 0.1.5). https://CRAN.R-project.org/package=rangeModelMetadata.

Kass, J.M.; Vilela, B.; Aiello-Lammens, M.E.; Muscarella, R.; Merow, C.; Anderson, R.P. Wallace: A flexible platform for reproducible modeling of species niches and distributions built for community expansion. *Methods in Ecology and Evolution* **2018**, *9*, 1151-1156.

Kass, J.M.; Pinilla-Buitrago, G.E.; Paz, A.; Johnson, B.A.; Grisales-Betancur, V.; Meenan, S.I.; Attali, D.; Broennimann, O.; Galante, P.J.; Maitner, B.S.; et al. wallace 2: a shiny app for modeling species niches and distributions redesigned to facilitate expansion via module contributions. *Ecography* **2023**, *2023*, e06547.

Xie, Yihui. 2014. "Knitr: A Comprehensive Tool for Reproducible Research in R." In *Implementing Reproducible Computational Research*, edited by Victoria Stodden, Friedrich Leisch, and Roger D. Peng. Chapman; Hall/CRC.

———. 2015. *Dynamic Documents with R and Knitr*. 2nd ed. Boca Raton, Florida: Chapman; Hall/CRC. https://yihui.org/knitr/.

———. 2024. *Knitr: A General-Purpose Package for Dynamic Report Generation in r* (version R package 1.48). https://yihui.org/knitr/.

Xie, Yihui, J. J. Allaire, and Garrett Grolemund. 2018. *R Markdown: The Definitive Guide*. Boca Raton, Florida: Chapman; Hall/CRC. https://bookdown.org/yihui/rmarkdown.

Xie, Yihui, Christophe Dervieux, and Emily Riederer. 2020. *R Markdown Cookbook*. Boca Raton, Florida: Chapman; Hall/CRC. https://bookdown.org/yihui/rmarkdown-cookbook.
