# Supplementary material for: Comparative Phylogeography of Two Specialist Rodents in Forest Fragments in Kenya
Source: Life (Basel). 2024 Nov 12;14(11):1469. doi: 10.3390/life14111469 (PMC11595787; doi:10.3390/life14111469)
Supplement: Supplementary file 1 [file life-14-01469-s001.zip › Supplementary File S3.pdf]

Supplementary

Comparative Phylogeography of Two Specialist Rodents in Forest Fragments in Kenya

Alois Wambua Mweu [1,2,†], Kenneth Otieno Onditi [1,2,3,*,†], Laxman Khanal [4], Simon Musila [2], Esther Kioko [2] and Xuelong Jiang [1,3,*]

[1] Key Laboratory of Genetic Evolution and Animal Models, Kunming Institute of Zoology, Chinese Academy of Sciences, Kunming 650201, China; aliwambua@gmail.com
[2] Zoology Section, National Museums of Kenya, Nairobi P.O. Box 40658-00100, Kenya
[3] Sino-Africa Joint Research Centre, Chinese Academy of Sciences, Nairobi P.O. Box 62000-00200, Kenya
[4] Central Department of Zoology, Institute of Science and Technology, Tribhuvan University, Kathmandu 44618, Nepal; lkhanal@cdztu.edu.np
* Correspondence: kenneth@mail.kiz.ac.cn (K.O.O.); jiangxl@mail.kiz.ac.cn (X.J.)
† These authors contributed equally to this work.

Supplementary File S3: Species morphological characterization

Supplemental Information 3 Comparison of external anatomical traits of *H. endoroabe* and *P. jacksoni* between forest fragments in Kenya. The values in each cell are in the form 'mean (min-max, SD)' where 'min' is the minimum value, 'max' the maximum value and 'SD' the standard deviation. 'N' represents the number of samples. See Figure 2 for the diagrammatic illustration.

H. endorobae						
Site	Weight	Head + body	Tail	Hind foot	Ear	N
Mau-Olpusimoru	22.98 (15.9-33.5, 4.41)	93.07 (84-106, 6.9)	138.07 (127-153, 7.6)	20.07 (19-21, 0.73)	19.36 (18-2, 0.63)	14
Mau-Trans-Mara	22.10 (22.1-22.1, NA)	93 (93-93, NA)	137 (137-137, NA)	19 (19-19, NA)	21 (21-21, NA)	1
MtKenya-Chogoria	22.43 (13.2-40.9, 5.95)	93.71 (78-115, 8.41)	139.39 (111-164, 11.63)	20.17 (18-23, 0.8)	18.39 (17-2, 0.9)	76
MtKenya-Sirimon	26 (13.5-44, 7.11)	98.29 (78-134, 9.68)	138.62 (109-165, 12.95)	20.51 (18.5-25, 1.01)	19.91 (17-22, 0.96)	170
P. jacksoni						
Site	Weight	Head + body	Tail	Hind foot	Ear	N
Kakamega	36.10 (23.6-51.1, 6.02)	112.55 (97-127, 6.79)	142.52 (12-16, 7.7)	24.08 (22-26, 0.91)	20.70 (19-23, 0.86)	100
Loita-Empurputial	32.52 (17.3-57.3, 6.92)	110.60 (9-126, 8.15)	133.09 (108-16, 11.39)	23.75 (21.5-26, 0.84)	21.77 (19.5-24.5, 0.99)	144

Loita-EmpurputiaII	37.39 (22.7-54.3, 7.71)	114.45 (10-129, 7.21)	134.76 (111-149, 11.19)	23.53 (22-26, 0.87)	21.64 (17-24, 1.41)	33
Loita-Entesekera	31.98 (20.1-42, 6.6)	111 (96-125, 8.1)	129.17 (116-147, 10.67)	23.25 (21-24.5, 1.08)	21.41 (2-23.5, 0.98)	12
Loita-Ilkimpa	35.84 (21.7-49, 6.99)	113.32 (93-126, 9.09)	135.84 (109-158, 11.09)	23.89 (22-26, 0.95)	21.87 (19-25, 1.12)	56
Loita-Olorte	37.80 (21.5-53.7, 6.27)	111.63 (93-127, 7.71)	141.39 (116-165, 8.68)	23.95 (22-26, 0.97)	21.87 (18.5-23.5, 0.77)	104
Mau-Chemorgok	36.43 (19.3-51.6, 7.03)	110.41 (92-129, 7.92)	136.87 (108-158, 11.02)	23.78 (2-26, 1.04)	21.79 (2-24, 0.98)	86
Mau-Esageri	37.86 (25.6-49.3, 5.93)	111.50 (97-126, 6.82)	137.79 (12-147, 6.86)	24.21 (21-26, 1.04)	21.97 (2-24, 0.91)	38
Mau-Kericho	40.09 (28.5-49.3, 6.13)	114.78 (102-125, 6.14)	136.18 (115-148, 6.41)	24.05 (22-25, 0.78)	22.05 (2-24, 0.9)	40
Mau-Olpusimoru	34.87 (24.9-47.9, 5.37)	110.42 (96-123, 6.3)	134.68 (123-147, 6.68)	23.64 (21-26, 1.1)	22.04 (2-24, 0.92)	50
Mau-Trans-Mara	42.85 (29.9-50.2, 5.28)	119.57 (107-129, 5.75)	136.82 (114-161, 7.1)	23.59 (21-25, 1.08)	22.63 (2-25, 1.03)	49
MtKenya-Chogoria	34.29 (12.6-57.4, 6.88)	109.46 (88-125, 7.37)	142.05 (116-165, 8.86)	22.92 (2-25, 1)	20.11 (17-22.5, 1.04)	157
MtKenya-Sirimon	34.33 (16.3-51.2, 7.26)	108.32 (82-125, 8.63)	127.32 (109-146, 8.97)	22.74 (19-25, 1.09)	20.69 (18-23, 1.05)	44

Sexual dimorphism – ANOVA tests – Combined

				Bootstrap[a]			
						95% Confidence Interval	
sex			Statistic	Bias	Std. Error	Lower	Upper
F	Mean	Weight	30.935	-.004	.366	30.212	31.652
		Head_body	104.957	.000	.466	104.045	105.883
		Tail	135.807	-.032	.663	134.390	137.034
		Hind_foot	22.435	.001	.077	22.286	22.583
		Ear	20.688	-.002	.067	20.556	20.819
	N	Weight	542	0	17	506	577
		Head_body	542	0	17	506	577
		Tail	542	0	17	506	577
		Hind_foot	542	0	17	506	577

		Ear	542	0	17	506	577
	Std. Deviation	Weight	8.4699	-.0057	.2800	7.9414	9.0595
		Head_body	10.8436	-.0039	.3287	10.1938	11.5041
		Tail	15.6344	-.0149	1.2438	13.3441	18.1725
		Hind_foot	1.7935	-.0025	.0552	1.6879	1.9027
		Ear	1.6051	-.0034	.0745	1.4689	1.7565
M	Mean	Weight	34.215	-.007	.340	33.574	34.886
		Head_body	109.160	-.004	.423	108.294	109.960
		Tail	135.936	.011	.497	134.975	136.923
		Hind_foot	23.060	-.002	.068	22.921	23.190
		Ear	20.983	-.001	.059	20.866	21.098
	N	Weight	662	-1	17	627	697
		Head_body	662	-1	17	627	697
		Tail	662	-1	17	627	697
		Hind_foot	662	-1	17	627	697
		Ear	662	-1	17	627	697
	Std. Deviation	Weight	9.0747	-.0160	.2265	8.6117	9.4927
		Head_body	11.2773	-.0311	.3549	10.5438	11.9196
		Tail	12.8943	-.0413	.5066	11.8597	13.8909
		Hind_foot	1.7544	.0000	.0463	1.6663	1.8458
		Ear	1.5602	-.0011	.0428	1.4748	1.6400
Total	Mean	Weight	32.742	-.008	.241	32.255	33.198
		Head_body	107.289	-.005	.309	106.715	107.889
		Tail	135.906	-.008	.394	135.141	136.672
		Hind_foot	22.786	-.002	.050	22.681	22.884
		Ear	20.850	-.002	.044	20.763	20.938
	N	Weight	1240	0	0	1240	1240
		Head_body	1240	0	0	1240	1240
		Tail	1240	0	0	1240	1240
		Hind_foot	1240	0	0	1240	1240
		Ear	1240	0	0	1240	1240
	Std. Deviation	Weight	8.9501	-.0061	.1614	8.6398	9.2752
		Head_body	11.2722	-.0120	.2282	10.8230	11.7009
		Tail	14.2473	-.0033	.6609	13.1197	15.5304
		Hind_foot	1.8010	-.0004	.0356	1.7352	1.8695
		Ear	1.5798	-.0010	.0400	1.5011	1.6627

			Sum of Squares	df	Mean Square	F	Sig.
Weight * sex	Between Groups	(Combined)	3207.639	2	1603.820	20.657	.000
	Within Groups		96041.014	1237	77.640		
	Total		99248.653	1239			
Head_body * sex	Between Groups	(Combined)	5284.345	2	2642.172	21.482	.000
	Within Groups		152145.258	1237	122.995		
	Total		157429.603	1239			
Tail * sex	Between Groups	(Combined)	36.837	2	18.419	.091	.913
	Within Groups		251460.623	1237	203.283		
	Total		251497.460	1239			
Hind_foot * sex	Between Groups	(Combined)	118.353	2	59.176	18.768	.000
	Within Groups		3900.303	1237	3.153		
	Total		4018.656	1239			
Ear * sex	Between Groups	(Combined)	25.868	2	12.934	5.218	.006
	Within Groups		3066.212	1237	2.479		
	Total		3092.080	1239			

Sexual dimorphism – ANOVA tests – *H. endorobae*

				Bootstrap[a]			
						95% Confidence Interval	
sex			Statistic	Bias	Std. Error	Lower	Upper
F	Mean	Weight	23.336	-.046	.525	22.221	24.302
		Head_body	94.574	-.049	.807	92.939	96.102
		Tail	134.208	.012	1.946	130.256	137.858
		Hind_foot	20.018	-.004	.089	19.824	20.182
		Ear	19.215	-.005	.127	18.948	19.447
	N	Weight	142	0	11	120	164
		Head_body	142	0	11	120	164
		Tail	142	0	11	120	164
		Hind_foot	142	0	11	120	164
		Ear	142	0	11	120	164
	Std. Deviation	Weight	6.2149	-.0507	.3632	5.4270	6.8332

		Head_body	9.5265	-.0569	.7092	8.2015	10.8555
		Tail	22.7442	-.4095	3.1105	16.3196	28.2929
		Hind_foot	1.0609	-.0139	.1342	.8183	1.3363
		Ear	1.5210	-.0146	.1934	1.2016	1.9190
M	Mean	Weight	24.555	-.003	.702	23.147	26.015
		Head_body	95.905	.005	.962	93.949	97.839
		Tail	132.720	-.017	1.459	129.786	135.682
		Hind_foot	20.477	.000	.090	20.306	20.652
		Ear	19.297	-.001	.099	19.112	19.497
	N	Weight	148	0	11	126	169
		Head_body	148	0	11	126	169
		Tail	148	0	11	126	169
		Hind_foot	148	0	11	126	169
		Ear	148	0	11	126	169
	Std. Deviation	Weight	8.5050	-.0497	.3933	7.6511	9.2734
		Head_body	11.6048	-.0723	.5440	10.4484	12.5994
		Tail	18.1499	-.1202	1.2585	15.6750	20.5970
		Hind_foot	1.1000	-.0106	.0905	.9178	1.2714
		Ear	1.2370	-.0081	.0708	1.0855	1.3728
NA	Mean	Weight	27.591	-.082	2.660	22.666	33.034
		Head_body	100.818	-.122	3.633	93.564	107.856
		Tail	139.545	.343	7.470	123.557	151.664
		Hind_foot	20.818	.003	.345	20.112	21.500
		Ear	19.818	.001	.303	19.200	20.400
	N	Weight	11	0	3	5	18
		Head_body	11	0	3	5	18
		Tail	11	0	3	5	18
		Hind_foot	11	0	3	5	18
		Ear	11	0	3	5	18
	Std. Deviation	Weight	8.8014	-.8044	2.1127	3.6733	11.6142
		Head_body	12.0401	-1.0282	2.6125	5.2197	15.8037
		Tail	24.5942	-3.4258	9.4128	7.2717	37.4900
		Hind_foot	1.1677	-.0796	.2280	.6162	1.5092
		Ear	.9816	-.0624	.1816	.5272	1.2535
Total	Mean	Weight	24.091	-.025	.449	23.164	25.000
		Head_body	95.457	-.024	.643	94.244	96.700
		Tail	133.671	.012	1.202	131.157	135.963

		Hind_foot	20.273	-.001	.062	20.146	20.389
		Ear	19.277	-.002	.078	19.117	19.427
	N	Weight	301	0	15	272	332
		Head_body	301	0	15	272	332
		Tail	301	0	15	272	332
		Hind_foot	301	0	15	272	332
		Ear	301	0	15	272	332
	Std. Deviation	Weight	7.5502	-.0329	.2865	6.9767	8.0932
		Head_body	10.7237	-.0388	.4255	9.8960	11.5757
		Tail	20.6532	-.1827	1.6926	17.2634	23.9220
		Hind_foot	1.1089	-.0063	.0799	.9559	1.2693
		Ear	1.3718	-.0056	.1070	1.1922	1.5926

ANOVA Table

			Sum of Squares	df	Mean Square	F	Sig.
Weight * sex	Between Groups	(Combined)	247.624	2	123.812	2.189	.114
	Within Groups		16854.082	298	56.557		
	Total		17101.706	300			
Head_body * sex	Between Groups	(Combined)	456.653	2	228.326	1.999	.137
	Within Groups		34042.786	298	114.238		
	Total		34499.439	300			
Tail * sex	Between Groups	(Combined)	554.477	2	277.238	.648	.524
	Within Groups		127412.462	298	427.559		
	Total		127966.939	300			
Hind_foot * sex	Between Groups	(Combined)	18.692	2	9.346	7.953	.000
	Within Groups		350.224	298	1.175		
	Total		368.917	300			
Ear * sex	Between Groups	(Combined)	3.832	2	1.916	1.018	.362
	Within Groups		560.754	298	1.882		
	Total		564.586	300			

Sexual dimorphism – ANOVA tests – *P. jacksoni*

sex			Statistic	Bootstrap Bias	Bootstrap Std. Error	Bootstrap 95% Confidence Interval Lower	Bootstrap 95% Confidence Interval Upper
F	Mean	Weight	33.632	.000	.371	32.910	34.346
		Head_body	108.643	-.015	.436	107.775	109.455
		Tail	136.375	-.030	.609	135.122	137.503
		Hind_foot	23.294	-.001	.054	23.185	23.392
		Ear	21.211	-.002	.064	21.088	21.329
	N	Weight	400	0	17	365	432
		Head_body	400	0	17	365	432
		Tail	400	0	17	365	432
		Hind_foot	400	0	17	365	432
		Ear	400	0	17	365	432
	Std. Deviation	Weight	7.4684	.0015	.3335	6.8573	8.1257
		Head_body	8.6818	-.0059	.3308	8.0370	9.3567
		Tail	12.1403	.0032	.4909	11.2017	13.1317
		Hind_foot	1.0699	-.0030	.0397	.9885	1.1428
		Ear	1.2763	-.0009	.0516	1.1739	1.3775
M	Mean	Weight	36.997	.000	.320	36.345	37.647
		Head_body	112.977	.006	.344	112.303	113.677
		Tail	136.862	.016	.482	135.891	137.792
		Hind_foot	23.804	.001	.048	23.711	23.895
		Ear	21.468	.000	.058	21.361	21.587
	N	Weight	514	0	17	482	546
		Head_body	514	0	17	482	546
		Tail	514	0	17	482	546
		Hind_foot	514	0	17	482	546
		Ear	514	0	17	482	546
	Std. Deviation	Weight	7.1211	-.0107	.2282	6.6572	7.5482
		Head_body	7.7459	-.0111	.2984	7.1360	8.3226
		Tail	10.7698	-.0113	.4125	9.9820	11.5813
		Hind_foot	1.0680	-.0035	.0416	.9883	1.1540
		Ear	1.2817	-.0023	.0467	1.1913	1.3678
Total	Mean	Weight	35.515	-.001	.248	35.056	36.004
		Head_body	111.082	-.005	.282	110.508	111.643
		Tail	136.622	-.005	.372	135.854	137.303
		Hind_foot	23.592	.000	.037	23.519	23.665

		Ear	21.354	-.001	.043	21.269	21.439
	N	Weight	939	0	15	909	968
		Head_body	939	0	15	909	968
		Tail	939	0	15	909	968
		Hind_foot	939	0	15	909	968
		Ear	939	0	15	909	968
	Std. Deviation	Weight	7.4743	.0015	.1851	7.1254	7.8464
		Head_body	8.4692	.0009	.2177	8.0542	8.8901
		Tail	11.3833	.0076	.3147	10.7853	12.0372
		Hind_foot	1.1020	-.0021	.0294	1.0418	1.1564
		Ear	1.2832	-.0003	.0330	1.2212	1.3467

ANOVA Table

			Sum of Squares	df	Mean Square	F	Sig.
Weight * sex	Between Groups	(Combined)	2549.492	2	1274.746	23.934	.000
	Within Groups		49852.498	936	53.261		
	Total		52401.990	938			
Head_body * sex	Between Groups	(Combined)	4225.728	2	2112.864	31.364	.000
	Within Groups		63054.957	936	67.366		
	Total		67280.686	938			
Tail * sex	Between Groups	(Combined)	78.085	2	39.043	.301	.740
	Within Groups		121467.703	936	129.773		
	Total		121545.788	938			
Hind_foot * sex	Between Groups	(Combined)	62.735	2	31.368	27.277	.000
	Within Groups		1076.388	936	1.150		
	Total		1139.124	938			
Ear * sex	Between Groups	(Combined)	14.921	2	7.460	4.565	.011
	Within Groups		1529.672	936	1.634		
	Total		1544.593	938			

Stat Table

Row Labels	Weight	Head-body	Min	Max	Tail	Min	Max	Hind-foot	Min	Max	Ear	Min	Max
Hylomyscus endorobae	24.1 (9.2-46.4)	**95.4**	**64.0**	**134.0**	**133.4**	**14.5**	**179.0**	**20.3**	**14.0**	**25.0**	**19.3**	**10.0**	**23.0**
**Female**	23.3 (9.2-39.9)	**94.6**	**70.0**	**134.0**	**134.4**	**14.5**	**160.0**	**20.0**	**14.0**	**24.0**	**19.2**	**10.0**	**22.0**
Mau	22.6 (18.6-26.4)	92.9	84.0	103.0	123.7	14.5	147.0	19.4	18.0	20.0	19.7	19.0	21.0
MtKenya	23.4 (9.2-39.9)	94.7	70.0	134.0	135.2	47.0	160.0	20.1	14.0	24.0	19.2	10.0	22.0
**Male**	24.6 (10.0-46.4)	**95.9**	**64.0**	**120.0**	**132.7**	**64.0**	**179.0**	**20.5**	**17.5**	**25.0**	**19.3**	**16.0**	**23.0**
Mau	24.9 (15.9-33.5)	97.3	87.0	106.0	139.3	129.0	144.0	20.8	20.0	21.0	19.3	19.0	20.0
MtKenya	24.5 (10.0-46.4)	95.9	64.0	120.0	132.5	64.0	179.0	20.5	17.5	25.0	19.3	16.0	23.0
Praomys jacksoni	35.4 (10.4-99.0)	**110.6**	**70.0**	**129.0**	**136.7**	**101.0**	**165.0**	**23.5**	**18.0**	**29.0**	**21.3**	**15.0**	**25.0**
**Female**	33.1 (10.4-70.4)	**107.8**	**70.0**	**129.0**	**136.4**	**101.0**	**165.0**	**23.2**	**18.0**	**26.0**	**21.1**	**15.0**	**25.0**
Kakamega	33.5 (23.6-44.2)	109.9	97.0	122.0	141.1	105.0	160.0	23.7	22.0	26.0	20.6	19.0	23.0
Loita	32.0 (14.0-57.3)	107.7	70.0	129.0	133.6	101.0	165.0	23.3	18.0	25.0	21.5	17.0	25.0
Mau	35.4 (16.4-70.4)	108.9	83.0	125.0	135.9	105.0	152.0	23.4	20.0	26.0	21.8	17.0	24.0
MtKenya	32.2 (10.4-57.4)	105.4	70.0	122.0	139.3	102.0	165.0	22.5	19.0	25.0	19.9	15.0	22.5
**Male**	37.2 (11.2-99.0)	**112.9**	**78.0**	**129.0**	**137.0**	**101.0**	**161.0**	**23.8**	**20.0**	**29.0**	**21.4**	**16.0**	**25.0**
Kakamega	39.1 (25.6-57.4)	115.2	103.0	127.0	143.3	129.0	160.0	24.4	22.0	26.0	20.8	19.0	23.0
Loita	36.7 (12.2-99.0)	112.6	83.0	129.0	135.7	101.0	159.0	23.9	20.0	29.0	21.7	16.5	24.0
Mau	39.8 (23.7-51.6)	115.0	94.0	129.0	136.8	109.0	161.0	24.0	21.0	29.0	22.2	19.0	25.0
MtKenya	33.8 (11.2-50.3)	109.4	78.0	125.0	136.6	101.0	159.0	22.9	20.0	25.0	20.2	16.0	23.0
