# Supplementary material for: Comparative Phylogeography of Two Specialist Rodents in Forest Fragments in Kenya
Source: Life (Basel). 2024 Nov 12;14(11):1469. doi: 10.3390/life14111469 (PMC11595787; doi:10.3390/life14111469)
Supplement: Supplementary file 1 [file life-14-01469-s001.zip › Supplementary File S5.pdf]

Supplementary

Comparative Phylogeography of Two Specialist Rodents in Forest Fragments in Kenya

Alois Wambua Mweu [1,2,†], Kenneth Otieno Onditi [1,2,3,*,†], Laxman Khanal [4], Simon Musila [2], Esther Kioko [2] and Xuelong Jiang [1,3,*]

[1] Key Laboratory of Genetic Evolution and Animal Models, Kunming Institute of Zoology, Chinese Academy of Sciences, Kunming 650201, China; aliwambua@gmail.com
[2] Zoology Section, National Museums of Kenya, Nairobi P.O. Box 40658-00100, Kenya
[3] Sino-Africa Joint Research Centre, Chinese Academy of Sciences, Nairobi P.O. Box 62000-00200, Kenya
[4] Central Department of Zoology, Institute of Science and Technology, Tribhuvan University, Kathmandu 44618, Nepal; lkhanal@cdztu.edu.np
* Correspondence: kenneth@mail.kiz.ac.cn (K.O.O.); jiangxl@mail.kiz.ac.cn (X.J.)
† These authors contributed equally to this work.

Supplementary File S5. Genus-level genetic distances between species units

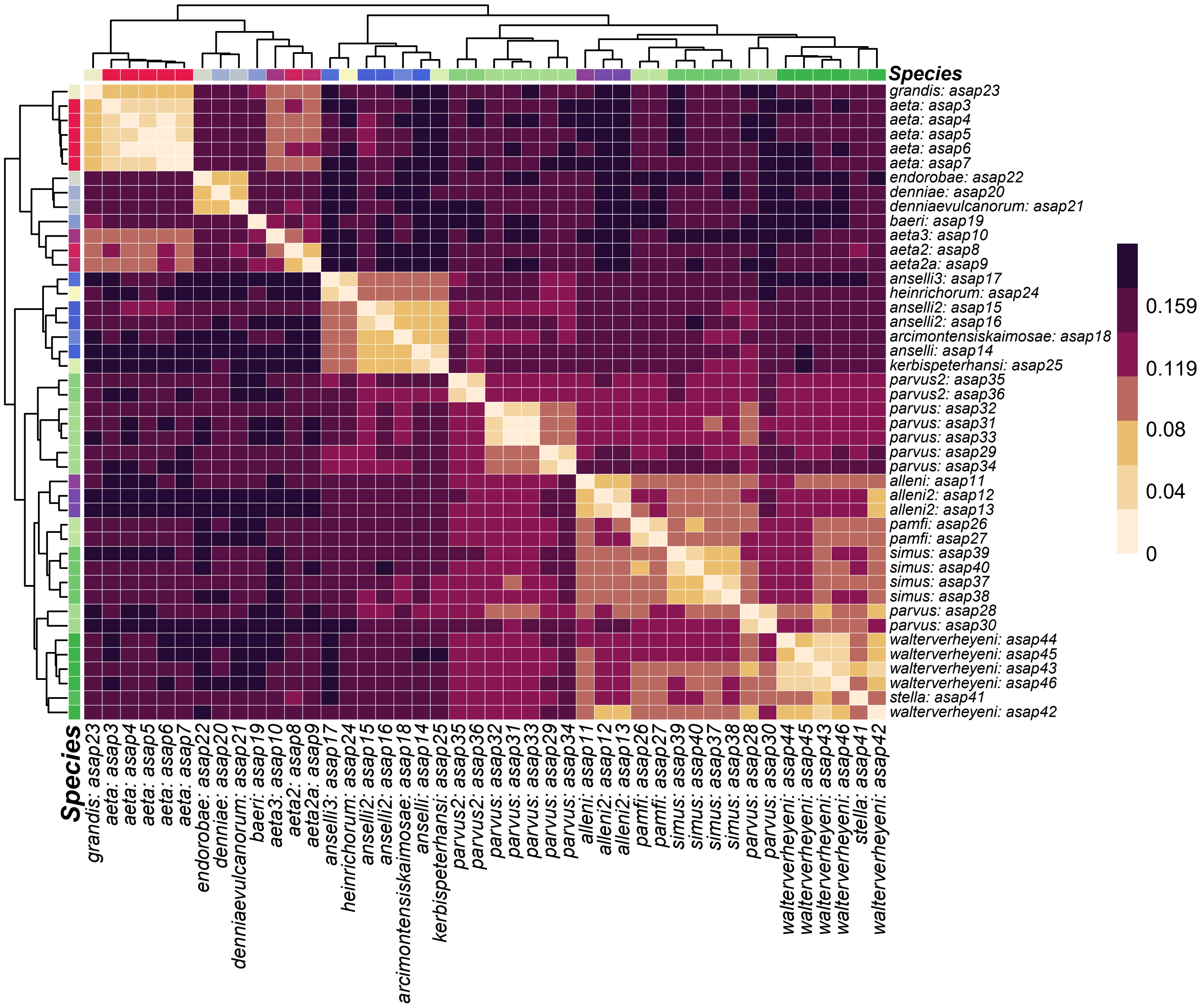

Species
grandis: asap23
aeta: asap3
aeta: asap4
aeta: asap5
aeta: asap6
aeta: asap7
endorobae: asap22
denniae: asap20
denniaevulcanorum: asap21
baeri: asap19
aeta3: asap10
aeta2: asap8
aeta2a: asap9
anselli3: asap17
heinrichorum: asap24
anselli2: asap15
anselli2: asap16
arcimontensiskaimosae: asap18
anselli: asap14
kerbispeterhansi: asap25
parvus2: asap35
parvus2: asap36
parvus: asap32
parvus: asap31
parvus: asap33
parvus: asap29
parvus: asap34
alleni: asap11
alleni2: asap12
alleni2: asap13
pamfi: asap26
pamfi: asap27
simus: asap39
simus: asap40
simus: asap37
simus: asap38
parvus: asap28
parvus: asap30
walterverheyeni: asap44
walterverheyeni: asap45
walterverheyeni: asap43
walterverheyeni: asap46
stella: asap41
walterverheyeni: asap42
0.159
0.119
0.08
0.04
0

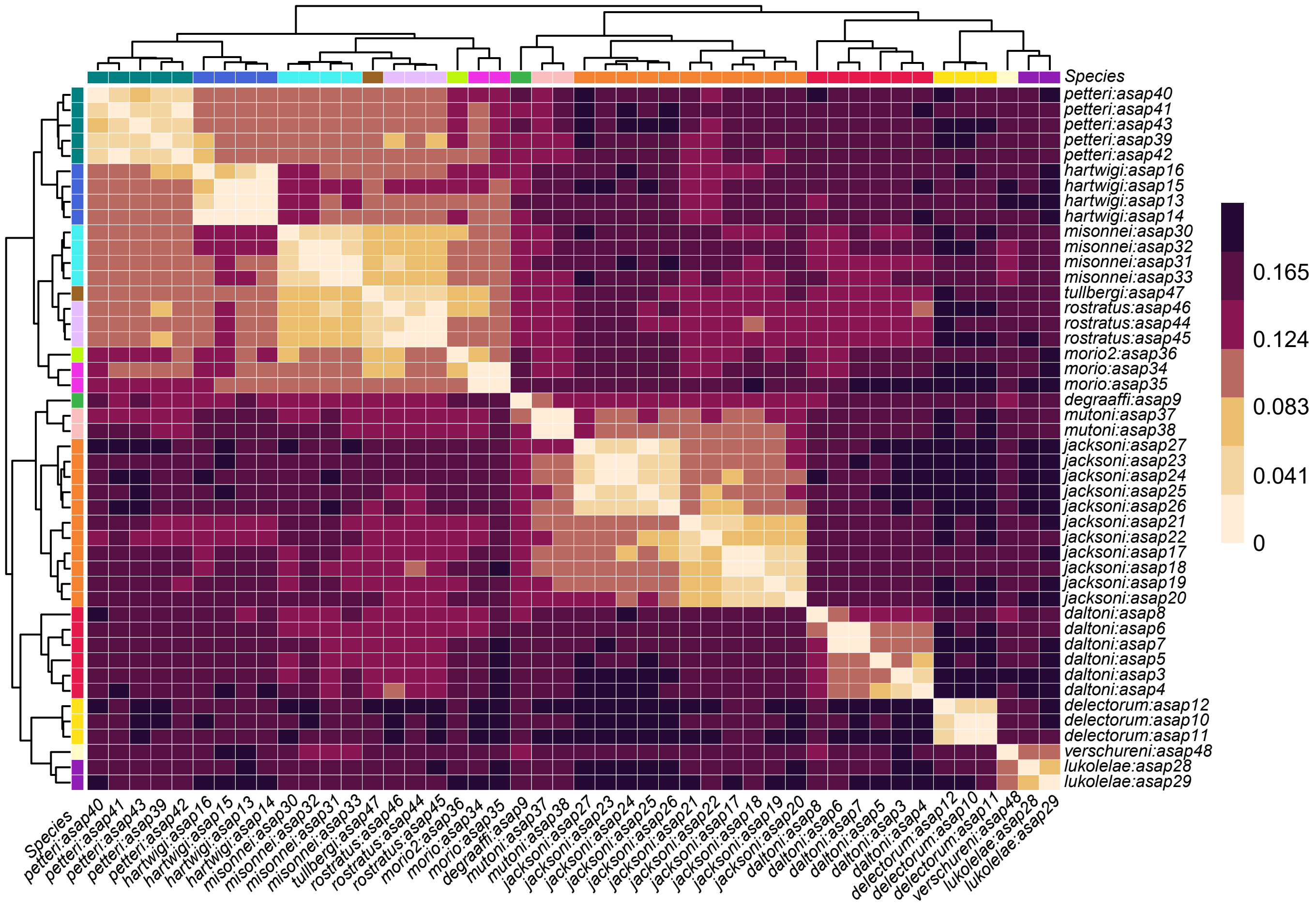
Species
petteri:asap40
petteri:asap41
petteri:asap43
petteri:asap39
petteri:asap42
hartwigi:asap16
hartwigi:asap15
hartwigi:asap13
hartwigi:asap14
misonnei:asap30
misonnei:asap32
misonnei:asap31
misonnei:asap33
tullbergi:asap47
rostratus:asap46
rostratus:asap44
rostratus:asap45
morio2:asap36
morio:asap34
morio:asap35
degraaffi:asap9
mutoni:asap37
mutoni:asap38
jacksoni:asap27
jacksoni:asap23
jacksoni:asap24
jacksoni:asap25
jacksoni:asap26
jacksoni:asap21
jacksoni:asap22
jacksoni:asap17
jacksoni:asap18
jacksoni:asap19
jacksoni:asap20
daltoni:asap8
daltoni:asap6
daltoni:asap7
daltoni:asap5
daltoni:asap3
daltoni:asap4
delectorum:asap12
delectorum:asap10
delectorum:asap11
verschureni:asap48
lukolelae:asap28
lukolelae:asap29
0.165
0.124
0.083
0.041
0
